# MiR-452 promotes an aggressive colorectal cancer phenotype by regulating a Wnt/β-catenin positive feedback loop

**DOI:** 10.1186/s13046-018-0879-z

**Published:** 2018-09-25

**Authors:** Tingting Li, Xiangyu Jian, Han He, Qiuhua Lai, Xianzheng Li, Danling Deng, Tengfei Liu, Jiehong Zhu, Hongli Jiao, Yaping Ye, Shuyang Wang, Minhui Yang, Lin Zheng, Weijie Zhou, Yanqing Ding

**Affiliations:** 1 Department of Pathology, Nanfang Hospital, Southern Medical University, Guangzhou, Guangdong China; 2 Department of Pathology, School of Basic Medical Sciences, Southern Medical University, Guangzhou, Guangdong China; 3 State Key Laboratory of Oncology in Southern China, Department of Experimental, Guangzhou, Guangdong China; 4 Department of Hematology, Nanfang Hospital, Southern Medical University, Guangzhou, Guangdong China; 5 Department of Gastroenterology, Nanfang Hospital, Southern Medical University, Guangzhou, Guangdong China; 6 Medical genetic center, Guangdong Women and Children Hospital, Guangzhou, Guangdong China; 7 Department of Pathology, Nanfang Hospital and School of Basic Medical Sciences, Southern Medical University, Guangzhou, 510515 China

**Keywords:** miR-452, Colorectal caner, Wnt/β-catenin pathway, GSK3β, TCF4/LEF1

## Abstract

**Background:**

Aberrant activation of Wnt/β-catenin signaling pathway is considered to be an important issue in progression and metastasis of various human cancers, especially in colorectal cancer (CRC). MiR-452 could activate of Wnt/β-catenin signaling. But the mechanism remains unclear.

**Methods:**

The expression of miR-452 in CRC and normal tissues was detected by real-time quantitative PCR. The effect of miR-452 on CRC growth and invasion was conducted by functional experiments in vitro and in vivo. Bioinformatics and cell luciferase function studies verified the direct regulation of miR-452 on the 3’-UTR of the GSK3β, which leads to the activation of Wnt/β-catenin signaling.

**Results:**

MiR-452 was upregulated in CRC compared with normal tissues and was correlated with clinical significance. The luciferase reporter system studies affirmed the direct regulation of miR-452 on the 3’-UTR of the GSK3β, which activate the Wnt/β-catenin signaling. The ectopic upregulation of miR-452 significantly inhibited the expression of GSK3β and enhanced CRC proliferation and invasion in vitro and in vivo. Meanwhile, knockdown of miR-452 significantly recovered the expression of GSK3β and attenuated Wnt/β-catenin-mediated cell metastasis and proliferation. More important, T-cell factor/lymphoid enhancer factor (TCF/LEF) family of transcription factors, which are crucial downstream molecules of the Wnt/β-catenin signaling pathway was verified as a valid transcription factor of miR-452’s promoter.

**Conclusions:**

Our findings first demonstrate that miR-452-GSK3β-LEF1/TCF4 positive feedback loop induce CRC proliferation and migration.

**Electronic supplementary material:**

The online version of this article (10.1186/s13046-018-0879-z) contains supplementary material, which is available to authorized users.

## Background

Human colorectal cancer (CRC) is one of the most common malignancies worldwide [1]. Although several kinds of treatment modalities have been developed recently for the patients with CRC, the clinical outcome of prognosis continues to be poor in patients with advanced CRC [1, 2]. Metastasis is responsible for the majority of cancer deaths [2]. The aberrant activation of Wnt/β-catenin signaling pathway is considered to be an important way in tumorigenesis and progression of CRC [3, 4]. Accumulation and nuclear localization of β-catenin is a hallmark of Wnt/β-catenin pathway activation [5, 6]. Upon activation of the Wnt signaling pathway, β-catenin escapes degradation and relocates to the nucleus where it cooperates with the T-cell factor/lymphoid enhancer factor (TCF/LEF) family of transcription factors to enable gene transcription downstream of Wnt pathway [7, 8].

MicroRNAs (miRNAs) are a class of noncoding regulatory RNA segments that bind to the 3′-untranslated region of specific mRNAs, leading to mRNA degradation or translation suppression. Recently, accumulating studies have reported that deregulation of miRNAs is essential for a series of human disease-related biological processes including cancer initiation, proliferation, apoptosis, angiogenesis, epithelial-mesenchymal transition (EMT), invasion and migration, etc. Several miRNAs (including miR-30b, miR-224, miR-17-5p, miR-214, miR-181b, miR-590-5p and miR-191) have been described as potential CRC biomarkers [9, 10]. MiR-452 is a member of the miR-224/miR-452 cluster. Our previous studies have validated that miR-224 is involved in human CRC tumor formation and progression by sustain Wnt/β-catenin pathway [10]. Recently, altered miR-452 expression has been reported in non-small cell lung cancer (NSCLC) [11, 12], glioma [13], urothelial carcinoma [14], prostate cancer [15, 16], osteosarcoma [17], and hepatocellular carcinoma [18, 19]. Nevertheless, little is known about the role of miR-452 and its regulation in CRC. The public database suggest us that miR-452 is highly expressed in colorectal cancer tissues. By bioinformatics predictions and functional analysis, we identified GSK3β as a direct downstream target of miR-452. As a crucial member of Wnt/β-catenin signaling modulators, GSK3β plays an important role in phosphorylation of β-catenin [6]. Downregulation of GSK3β could result in the accumulation of β-catenin in the nucleus and the perpetual transcription of Wnt target genes [7]. So we supposed that positive effect of miR-452 on proliferation and migration of human CRC cells could be manifested through the nuclear translocation of β-catenin and subsequently upregulation of its transcriptional targets c-Myc [7] and CyclinD1 [8].

In this study, we demonstrated that miR-452 plays an important role in regulating the Wnt/β-catenin signaling pathway by directly targeting GSK3β. We showed the positive effect of miR-452 on CRC cell’s tumorigenesis and aggressive phenotype. TCF/LEFs which are the high-mobility group (HMG) DNA-binding proteins with multiple domains for protein interaction and regulation, most closely associated with Wnt/β-catenin action. Meanwhile, we found the nuclear mediators TCF/LEFs which are most closely associated with Wnt/β-catenin could activate the promoter of miR-452. Our results first indicate that miR-452-mediated miR-452-GSK3β-LEF1/TCF4 positive feedback loop can induce CRC proliferation and migration.

## Methods

### Tissue specimens and cell cultures

We collected the colorectal cancer tissues from the Southern Medical University Institutional Board (Guangzhou, China) with prior approval. All samples were collected and analyzed with the prior written informed consent of the patients. All the CRC tissue samples and their matched adjacent normal tissues were collected at the operation room, Nanfang Hospital Southern Medical University, from 2008 to 2013. All the above tissue biopsies were frozen in liquid nitrogen until further use.

Human CRC cell line SW480 was cultured in RPMI1640 medium (Gibco, Grand Island, NY, USA) containing 5% fetal bovine serum (FBS; PAA Laboratories, Pasching, Austria); HCT116, HCT15 and SW620 cells were cultured in Dulbecco’s modified Eagle’s medium (DMEM; Gibco) supplemented with 5% FBS (PAA). All these cell lines were purchased from American Type Culture Collection Cell Biology Collection and were maintained in Department of Pathology, Southern Medical University.

### RNA extraction and real-time PCR

MirVana miRNA Isolation Kit (Ambion, Austin, TX, USA) was used in extracting total miRNA according to the manufacturer’s instructions. We synthesized cDNA from total RNA using the Taqman miRNA reverse transcription kit (Applied Biosystems, Foster City, CA, USA). Real-time PCR was performed using the Applied Biosystems 7500 Sequence Detection system, IQTM SYBR Green Supermix (BioRad Laboratories, Hercules, CA, USA) with 5 ng cDNA and 10pM of each primer. The cycling conditions and the method to calculate the expression of miRNA as described before [20]. Primer sequences were showed in Additional file 1: Figure S2.

### Plasmids and transfection

To generate a miR-452 expression vector, a 500 bp genomic fragment covering the region coding for pri-miR-452 and its downstream and upstream regions were PCR amplified and subsequently cloned into the pLvthm vector (Addgene). The full-length of GSK3β 3’-UTR is 4849 bp long. The miR-452 binding site in the GSK3β3’-UTR is located at 5917 to 5924 bp. The region of the human GSK3β 3’-UTR from 5857 to 6318 bp was generated by PCR amplification and subcloned into the Sac I/Xma I sites of the pGL3-basic luciferase reporter plasmid (Promega). The miR-452 mimics, negative control, and anti-miR-452 inhibitors were purchased from Genecopoeia (Genecopoeia Co. Ltd), and transfected into colorectal cancer cells using Lipofectamine 2000 reagent (Invitrogen) according to the manufacturer’s instructions.

### Western blotting

Western blot was performed as previous study described [9] using anti-GSK3β, anti-cyclinD1 (BD Pharmingen, Franklin Lakes, NJ, USA), anti-p-β-catenin, anti-β-catenin (Cell Signaling Technology,Danvers, MA,USA), anti-c-Myc and anti-MMP7 (Bioworld Technology, St. Louis Park, MN, USA). An anti-α-tubulin monoclonal antibody (Sigma, St. Louis, MO, USA) was used as a loading control [9].

### MTT assay, colony formation assay, wound-healing assay, transwell invasion assay

The miR-452 mimics, anti-miR-452 inhibitors and the control oligos were transiently transfected into CRC cells for the MTT assay, colony-formation assay, wound-healing assay, and transwell invasion assay.

Cells prepared for MTT assay were seeded on 96-well plates (1 × 10^3^ cells/well) to incubate for 24 h, 48 h, 72 h, 96 h, 120 h, and 148 h. On days one through six, 10ul of 5 mg/ml thiazolyl blue tetrazolium bromide solution (MTT, Sigma-Aldrich Chemical Company, St Louis MO, USA) were added in each well and incubated for 4 h. Afterwards, the medium supernatant was removed, and 100ul of dimethyl sulfoxide (DMSO Thermo Fisher Scientific, Waltham, MA, USA) was added in each well, incubating for 10 min. The optical density (OD) value (at 490 nm) was measured by the microplate reader. The growth curves were composed according to the OD values.

The colony formation assay is conducted in a 6-well plate in which cells were seeded (2 × 10^2^ cells/well) and cultured for 2 weeks. The colonies were stained with 1% crystal violet for 1 h after fixation with 5% paraformaldehyde for 10 min. The number of colonies, defined as > 50 cells/colony were counted.

Cells were seeded (5 × 10^5^ cells/well) in six-well plates for the wound-healing assay, and waiting for them to grow to confluence. Then a cell-free area was scratched with a p200 pipette tip in the middle of the cell monolayer in the plate. PBS was used to remove floating cells and fresh media was added. The cells were induced to migrate towards the gap. Capture images of migration into the cell-free area at regular intervals and comparing the migration rate of different cell lines. The time course of gap closure were calculated at 0 h, 24 h, 48 h, 96 h by using ImageJ software.

The cells (3 × 10^4^ cells/well) were seeded on the upper layer of transwell plates (Corning Incorporated, NY, USA) with 8 μm pore membrane in serum-free medium. The chambers had been precoated with matrigel according to the protocol (BD, Basement Membrane Matrix, Franklin Lakes, NJ, USA). The transwell chambers were putting in the 24-well plates with 10% serum medium for 48 h. The cells migrated through the polycarbonate membrane were stained with 1% crystal violet for 1 h after fixation with 5% paraformaldehyde for 10 min. The cells in lower compartment of the chamber that had invaded to the basal side of the membrane were counted using a light microscope in 5 random visual fields (× 200).

All of the above-mentioned experiments were performed three times independently. The data was calculated using unpaired t test.

### Xenograft model in nude mice

For tumourigenesis assays, we engineered SW480 cells to stably express high miR-452 and low miR-452 respectively, using a Plasmid-based system. Engraft tumors were generated by subcutaneous injection of CRC cells (2 × 10^6^), including SW480/Vector, SW480/miR-452, SW480/NC and SW480/ miR-452 inhibitor, into the axilla and groin of 4–6 week-old Balb/C athymic nude mice (nu/nu; Animal Centre of Southern Medical University, Guangzhou, China; *n* = 6 for each group). All mice were housed and sustained under specific pathogen-free conditions, and all experiments were approved by the Use Committee for Animal Care and performed in accordance with institutional guidelines. Tumor size was measured using a slide caliper and tumor volume was determined by the formula: 0.44 × A × B^2^, where A represents the diameter of the base of the tumor and B represents the corresponding perpendicular value. After euthanasia, the tumors were excised, fixed in 10% neutral buffered formalin, embedded in paraffin, and 4 μm sections were prepared and stained with haematoxylin.

### Orthotopic mouse metastatic model

A surgical orthotopic transplantation mouse model of CRC was performed as described before [21]. Cells (2 × 10^6^ per mouse) were subcutaneously injected into the right flank of 4-to 6-week-old Balb/C athymic nude mice (nu/nu) gained from the Animal Center of Southern Medical University, Guangzhou, China. The mice were killed after two weeks, and the tumors were excised and cut into small fragments approximately 1 mm in diameter. Nude mice were anesthetized and then transplanting the CRC tumor fragments to the cecum. All mice were kept in a highly sterile environment. The mice were killed 2 months after surgery, and the individual organs were excised and metastases were observed by staining with hematoxylin and eosin (H&E). The numbers of gross metastatic foci were observed using a dissection microscope. All animal experiments were conducted in accordance with standard procedures and approved by the institutional Use Committee for Animal Care.

### Immunohistochemistry

The CRC tissues soaked in formalin was embedded in paraffin and cut into 0.25um thin slices beard on glasses, dewaxed by dimethylbenzene and alcohol. Epitope/antigen retrieval was performed by boiling the tissues in citrate buffer (pH = 6.0) 5 min, then the tissues was put in horseradish peroxidase for 10 min after washed in PBS. BSA (bovine serum albumin) as blocking buffer incubated the tissues for 40 min. Primary antibody β-catenin, c-Myc, cyclinD1 and secondary antibody (ZSGB-BIO, Beijing, China) were used following the manufacturer’s instructions. After all staining is completed, sealing the stained sample by mounting a coverslip (Additional file 2).

### Luciferase assays

SW480 and HCT116 cells were seeded in triplicate respectively in 24-well plates (1 × 10^5^/ well) and cultured for 24 h. Luciferase reporter plasmids (100 ng) or control luciferase plasmid (100 ng) with pRL-TK Renilla plasmid (1 ng, Promega) were transfected into the cells using Lipofectamine 2000 (Invitrogen). Luciferase and Renilla signals were determined 24h after transfection via a Dual Luciferase Reporter AssayKit (Promega). Further details were described in our previous study [9].

### Chromatin immunoprecipitation assays

Sufficient chromatin which had been sheared to a size of 200-1500 bp, was generated via the method of fixation and sonication shearing of cells (approximately 1.5 × 10^7^). Then ChIP reactions were set up by adding the sheared chromatin with components like magnetic beads, antibody (LEF-1,TCF-4,IgG) and other components provided in kit (ChIP-IT® Express Magnetic Chromatin Immunoprecipitation Kit). After washing magnetic beads, chromatin was eluted, reversing cross-links and treated with proteinase K, so that the samples should be subjected to a DNA clean-up step prior to next real-time PCR. Real-time PCR for target was as previously introduced, and the primers used were summarized in Additional file 3: Figure S3.

### Statistical analysis

All statistical analyses were performed using SPSS19.0 for Windows (IBM, Armonk, NY, USA). The two-tailed paired Student’s t-test was used for analyzing two groups. The Mann-Whitney U-test and Spearman’s correlation analyses were used to analyze the relationship betweenmiR-452 expression and the clinicopathological features of CRC. The Pearson correlation analysis was used to analyze the relationship between miR-452 expression and GSK3β expression. Survival curves were plotted by the Kaplan-Meier method and compared with the log-rank test, *p* < 0.05 was considered statistically significant. All experiments were performed at least twice. Results are expressed as mean ± S.E.M. where applicable.

## Results

### MiR-452 is overexpressed in CRC

**Fig. 1:**
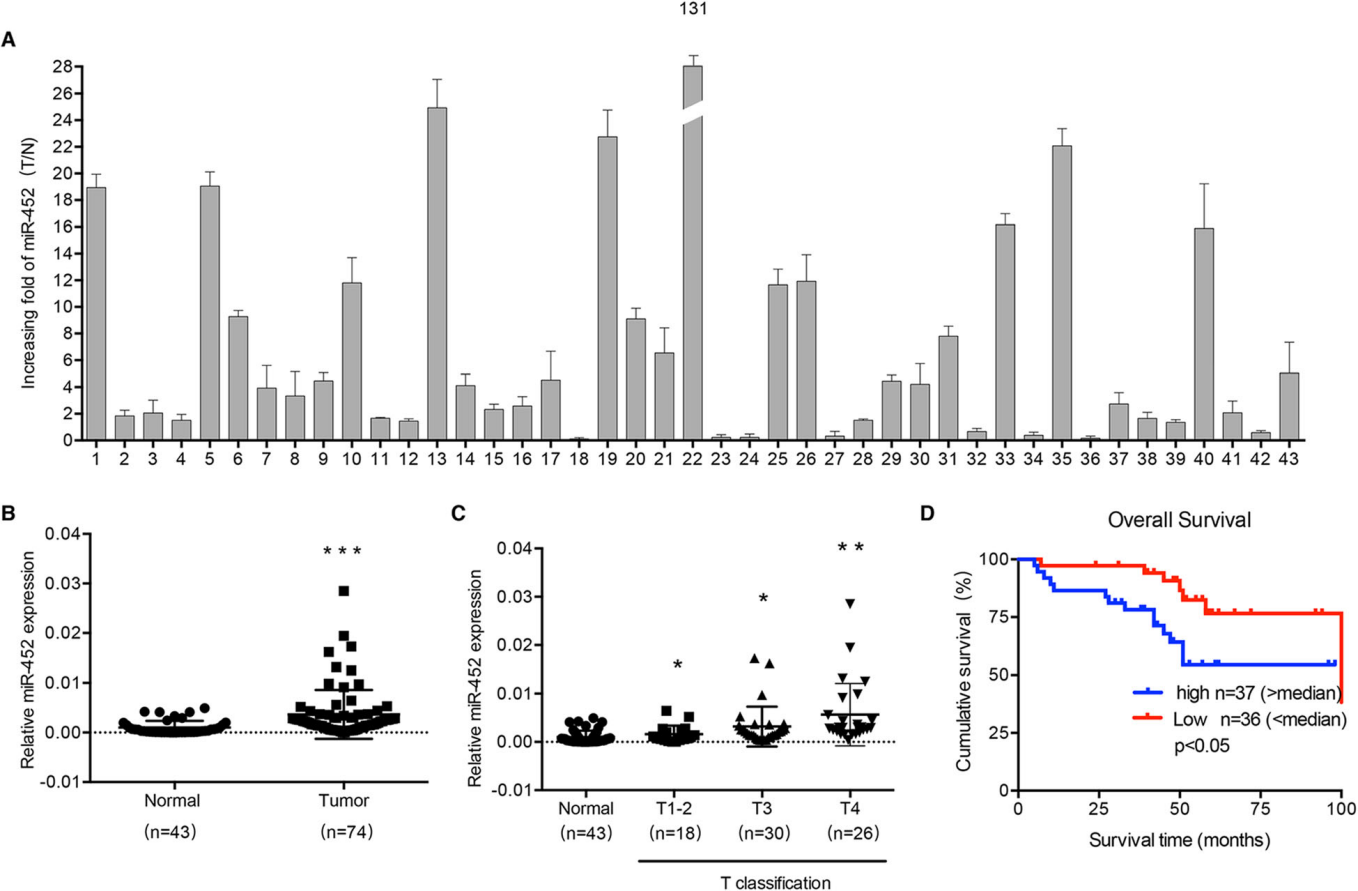
Expression of miR-452 in colorectal cancer. **a**, real-time quantitative PCR analysis of miR-452 expression in 43 paired human CRC tissues and their corresponding adjacent normal tissues; miR-452 expression was normalized to that of U6. **b**, relative expression of miR-452 in 43 normal and 74 CRC tissues. The Mann-Whitney test was used (*p* < 0.0001). **c**, miR-452 expression in normal colorectal tissues relative to CRC tissues with a stage 1–2 clinical classification; Student’s t-test was used (*p* < 0.05). **d**, correlation between miR-30b expression and distant metastasis. Correlation between miR-452 expression and survival by Kaplan-Meier analysis of CRC patients with high (≥ median; *n* = 37) or low (< median; *n* = 36) miR-452 expression (*p* < 0.05)

The expression of miR-452 was examined in 43 matched pairs of CRC and para-carcinoma normal tissues by real-time PCR. This analysis revealed that miR-452 was overexpressed in 74.4% (32/43) of the CRC tissue samples (T/*N* > 1.5-fold) compared to the matched adjacent normal tissues from the same patient (Fig. 1a). Student’s t-test revealed that the miR-452 expression levels were significantly lower in the adjacent normal tissues than in the CRC tissue samples (*p* < 0.05, Fig. 1b). In addition, miR-452 was expressed at relatively low levels in tumors with an early T classification (T1, T2 and T3), and at markedly increased levels in T4 tumors (Fig. 1c). Survival rates were higher in the patients who had low expression of miR-452 (Fig. 1d). Clinicopathologic statistics showed a correlation between miR-452 expression and CRC tissues (Table 1). Real-time PCR analysis was performed to detect the expression of miR-452 in 10 CRC cell lines including SW480, SW620, HCT116, HCT15, HT29, Caco2, DLD1, M5 Colo205 and Ls174t. MiR-452 expression was relatively high in SW620, while low expression was observed in SW480 (Additional file 4: Figure S1A).

**Table 1:** Correlation between clinicopathologic features and MiR-452 expression in 74 colorectal cancer tissues

Characteristics	miR-452 expression		*P* value
	Low	High	
Age			
< 63.5	19	18	0.816
> 63.5	18	19	
Gender			
Male	25	22	0.469
Female	12	15	
T classification			
1–2	13	5	0.030
3–4	24	32	
N classification			
0	21	23	0.636
1–2	16	14	
Distant metastasis			
No	29	32	0.359
Yes	8	5	
Pathologic stage			
1–2	19	24	0.239
3–4	18	13	

### MiR-452 directly targets the Wnt signaling suppressor GSK3β

**Fig. 2:**
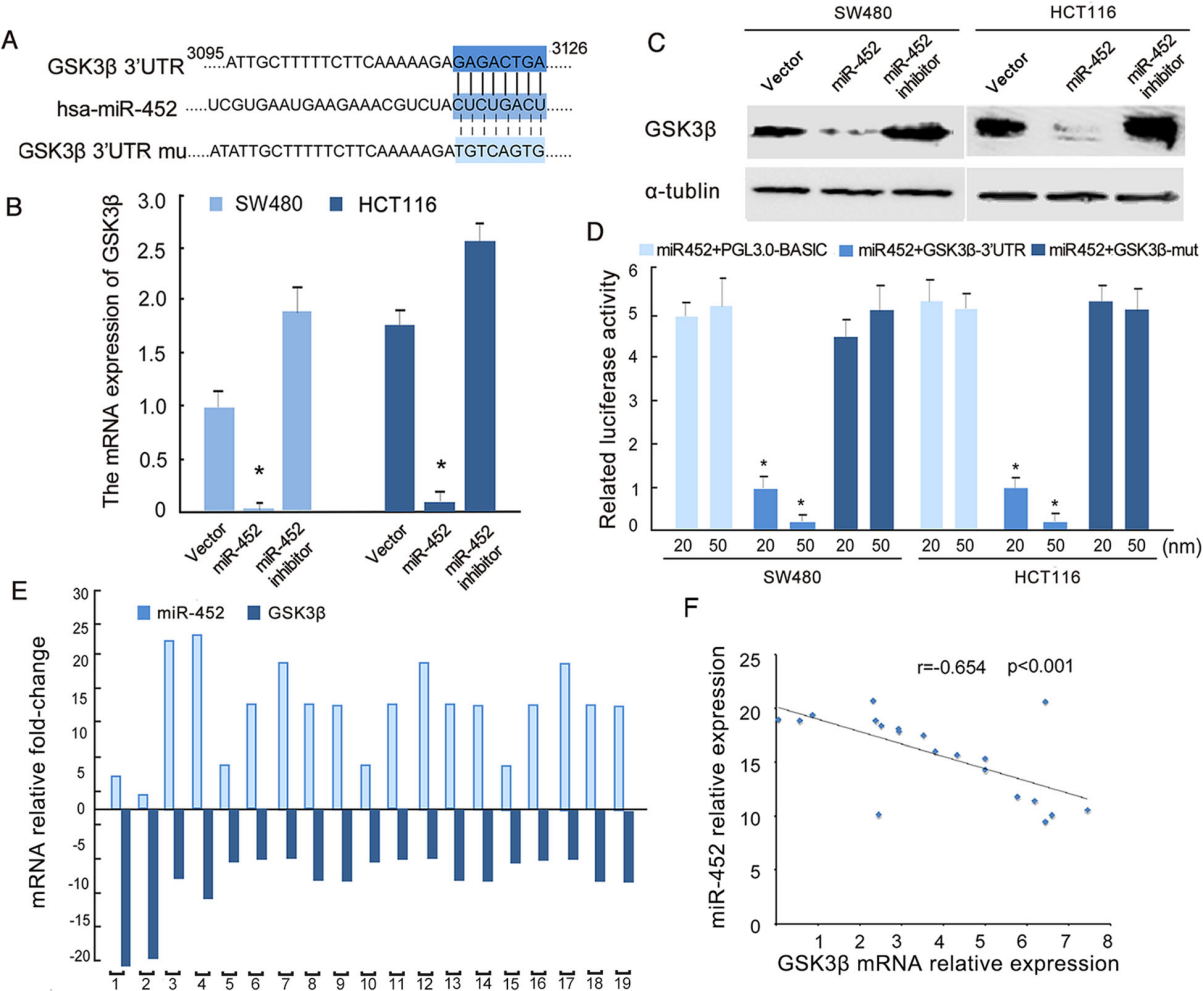
MiR-452 directly targets the 3’UTRs of GSK3β. **a**, predicted miR-30b target sequences in the 3’UTRs of GSK3β. The nucleotide mutants altered in the 3’UTRs of GSK3β are highlighted in light blue. **b**, real-time quantitative PCR analysis of GSK3β in the indicated cells. **c**, western blot analysis of GSK3β protein expression in the indicated cells. **d**, co-transfection of miR-452 mimic/indicated reporter gene in SW480 and HCT116 cells, luciferase activity assay expression. The error bars represent mean ± SD from three independent experiments. **e**, real-time quantitative PCR analysis of miR-452 and GSK3β expression in 19 human CRC tissues. The adjacent columns in different colors represent the relative expression levels of miR-452 and GSK3β in the same fresh CRC tissue. **f**, Spearman correlation btween miR-452 and GSK3β (*p* < 0.001)

Our previous study showed that the other member of the miR-224/miR-452 cluster, miR-224, could continuously activate Wnt signaling by directly targeting the 3’-UTR of GSK3β [10]. We hypothesized that GSK3β might also be a miR-452 target gene. Gene database analysis showed that the 3’-UTR of GSK3β, a classical negative regulator of Wnt signaling, contains a complementary site for the seed region of miR-452 (Fig. 2a). Real-time PCR (Fig. 2b) and western blot analysis (Fig. 2c) showed that both the mRNA and protein levels of GSK3β were significantly downregulated in miR-452-overexpressing cells. We then individually subcloned GSK3β 3’-UTR wild-type and mutant fragments into the pGL3-basic luciferase reporter vector. As shown in Fig. 2d, wild-type GSK3β reporter gene luciferase activity was reduced when miR-452 was overexpressed in both CRC cell lines. Next, we analyzed 19 fresh CRC tissue samples to explore the relationship between miR-452 and GSK3β. Figure 2e shows that miR-452 was upregulated while GSK3β was downregulated in CRC tissues. Spearman correlation analysis showed that miR-452 expression negatively correlates with expression of GSK3β (*r* = − 0.654, *p* < 0.001) (Fig. 2f).

### MiR-452 is required for Wnt/β-catenin signaling activation

**Fig. 3:**
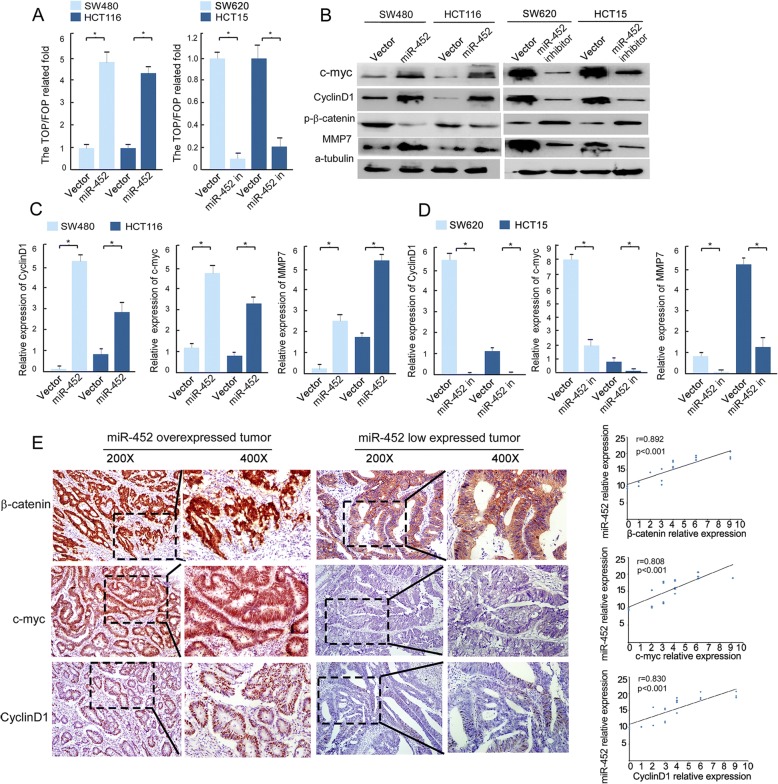
MiR-452 activated Wnt/β-catenin signaling pathway. **a**, the results of top/fop in indicated cells. **b**, western blot analysis of proteins produced downstream of Wnt signaling activation in the indicated cells. **c**, **d**, real-time quantitative PCR analysis of mRNA expression downstream of Wnt signaling activation in the vector and miR-452 cell lines (**c**) or in the vector and miR-452-inhibitor cell lines (**d**). The error bars represent the mean ± SD from three independent experiments. **e**, results of immunohistochemistry in human CRC tissues. The tumor and normal sections were stained using antibodies against β-catenin, c-myc and cyclin D1

As described above, miR-452 promotes the aggressive phenotype of CRC via direct binding to the 3’-UTR of GSK3β, a Wnt signaling suppressor. This prompted us to explore the role of miR-452 in Wnt signaling. The TOP/FOP luciferase assay demonstrated that the activity of the Wnt/β-catenin signaling pathway was significantly increased in miR-452-overexpressing CRC cells compared to control cells (Fig. 3a). In addition, we carried out western blot analysis and real-time PCR to investigate the direct or indirect downstream target genes of Wnt/β-catenin signaling (Fig. 3b, c, d). Figure 3c and d showed that overexpression of miR-452 significantly upregulated c-Myc, cyclinD1, MMP7, while inhibition of miR-452 clearly downregulated c-Myc, cyclinD1, MMP7. On the contrary, the phospho-β-Catenin protein level is decreased (Fig 3b). The protein and mRNA expression levels of total β-catenin are not affected (Additional file 4: Figure S1C and D). While the immunohistochemical staining showed that overexpression of miR-452 promotes nuclear relocalization of β-catenin and the expression of c-Myc and cyclinD1 (Fig. 3e left), which indicates that miR-452 promotes Wnt/β-catenin signaling pathway activity. Spearman correlation analysis showed that miR-452 expression positively correlates with expression of c-Myc and cyclinD1 and nuclear β-catenin (Fig. 3e right).

### Overexpression of miR-452 enhances proliferation and invasion capacity of CRC cells

**Fig. 4:**
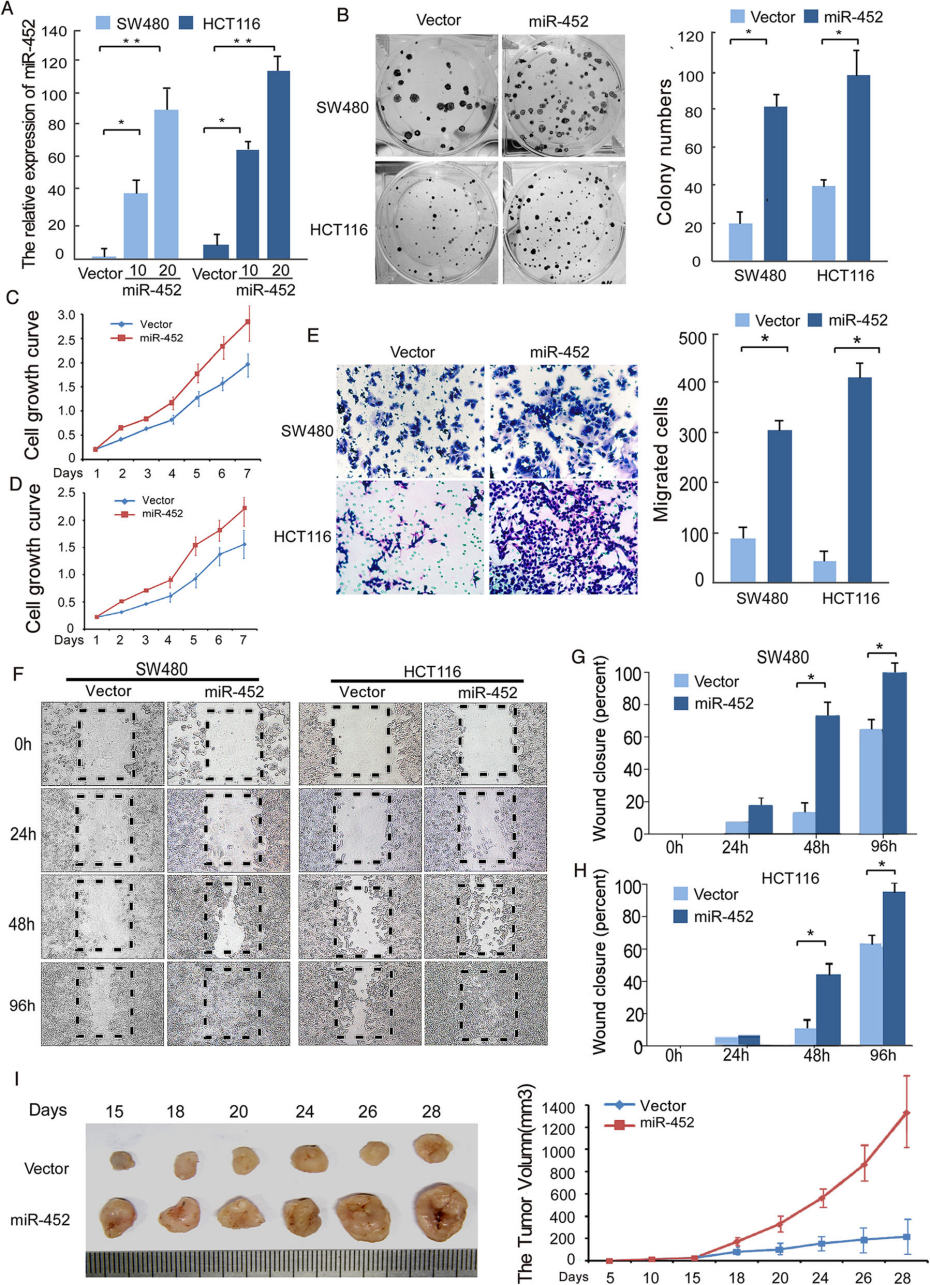
MiR-452 promoted the proliferation and invasiveness of CRC. **a**, real-time quantitative PCR analysis was used to measure expression of miR-452 in SW480 and HCT116 CRC cell lines transfected with different amounts of has-miR-452 mimics (10 and 20 nM). **b**, typical images from the colony formation assay; every well contained the number of cells> 50 cells. The colony count was of an entire well and the error bars represent mean ± SD from three independent experiments. **c**, **d**, the MTT assay showed divergent cell growth between SW480-Vector and SW480-mimic (**c**), or between HCT116-vector and HCT116-mimic (**d**). **e**, more cells transfected across the Matrigel-precoated membrane in cell lines treated with miR-452 mimics. **f**, **g**, **h**, representative results of the wound-healing assay in a fixed location at 4 regular intervals. **i**, tumor xenograft model. SW480-vector and SW480-miR-452 mimic cells were injected into the hindlimbs of nude mice (*n* = 6). Data points are presented as the mean tumor volume ± SD

We transfected the CRC cell lines SW480 and HCT116 with hsa-miR-452 mimic oligonucleotides successfully (Fig. 4a) and examined the effects on cellular proliferation. MTT and colony formation assays revealed that overexpression of miR-452 significantly increased the growth rate of both CRC cell lines compared to negative control transfected cells (Fig. 4b-d). Next, we utilized transwell chamber and wound healing assays to examine the effect of miR-452 on CRC cell invasion and migration. As shown in Fig. 4e-h, overexpression of miR-452 enhanced the migration and invasion capacity of both CRC cell lines compared to control cell lines. To confirm this effect in vivo, we engineered SW480 cells to stably overexpress miR-452 and performed a tumorigenesis experiment in nude mice. As shown in Fig. 4i, tumors in the SW480-miR-452 group grew more quickly than those in the SW480-Vector control group.

### Inhibition of miR-452 reduces the proliferation and invasion capacities of CRC cells

**Fig. 5:**
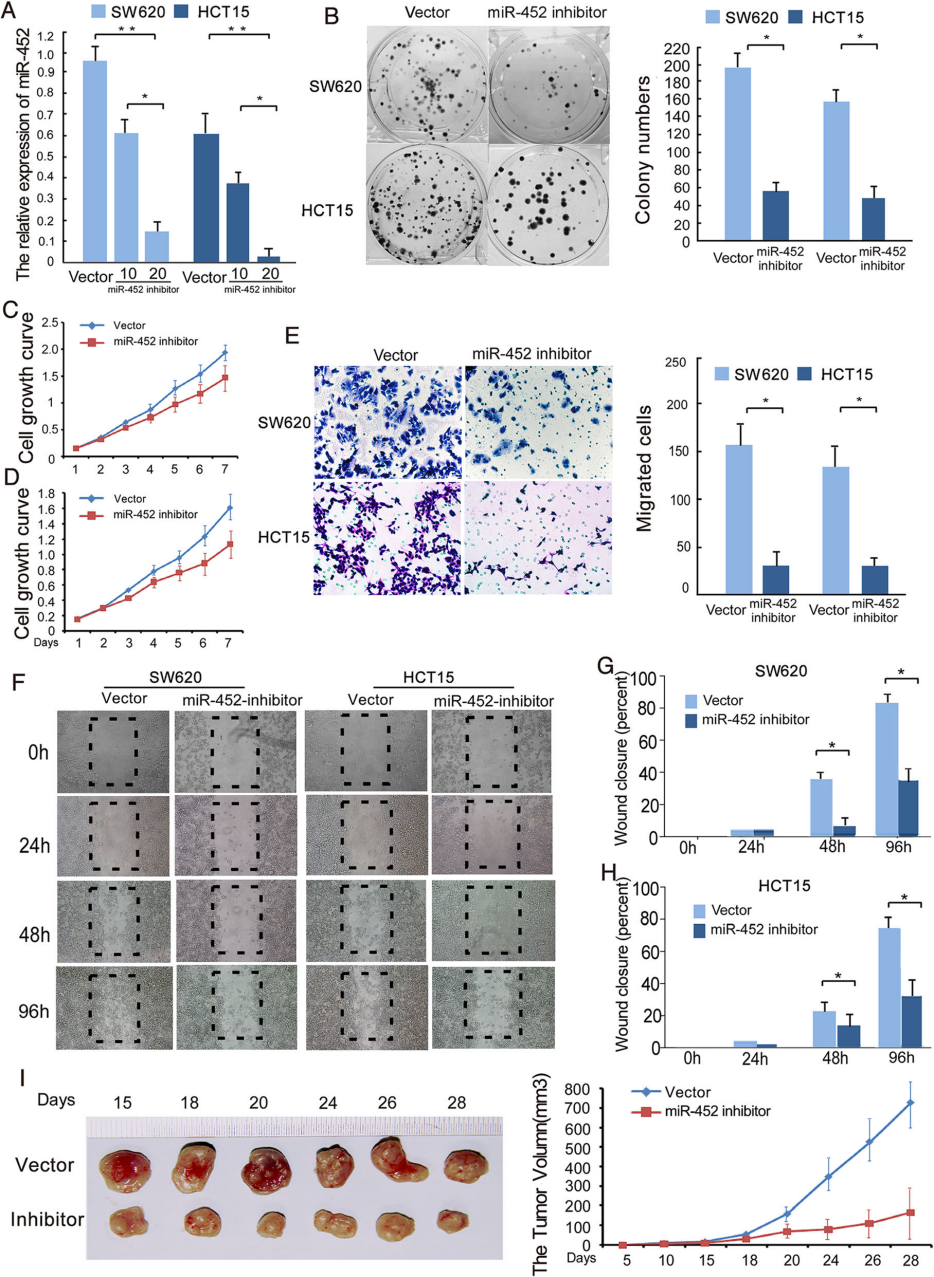
Inhibition of miR-452 reduced the proliferation and invasiveness of CRC. **a**, real-time quantitative PCR analysis was used to measure expression of miR-452 in SW620 and HCT15 CRC cell lines transfected with different amounts has-miR-452 inhibitor (10 and 20 nM). **b**, typical images from the colony formation assay; every well contained > 50 cells. The colony count was of an entire well and the error bars represent mean ± SD from three independent experiments. **c**, **d**, the MTT assay showed divergent cell growth between SW620-NC and SW620-inhibitor (**c**), or between HCT15-NC and HCT15-inhibitor (**d**). **e**, fewer cells transfected across the Matrigel-precoated membrane in cell lines treated with a miR-452 inhibitor. **f**, **g**, **h**, representative results of the wound-healing assay in a fixed location at 4 regular intervals. **i**, tumor xenograft model. SW620-NC and SW620-miR-452 inhibitor cells were injected into the hind limbs of nude mice (*n* = 6). Data points are presented as the mean tumor volume ± SD

We next suppressed miR-452 in SW620 and HCT15 cells by expressing miR-452 inhibitors (Fig. 5a) and explored the effects of miR-452 inhibition on cellular proliferation via MTT and colony formation assays. The results revealed that silencing miR-452 expression significantly decreased the growth rate of both CRC cell lines compared to negative control transfected cells (Fig. 5b-d). We utilized transwell chamber and wound healing assays to investigate whether inhibition of miR-452 suppresses the invasion and migration abilities of CRC cell lines. As shown in Fig. 5e-h, downregulated expression of miR-452 weakened the migration and invasion capacities of both CRC cell lines compared to control cell lines. To further confirm this effect in vivo, we constructed SW620 cells to stably express low levels of miR-452 and performed a xenograft model in nude mice. As shown in Fig. 5i, the tumors in the SW620-miR-452-inhibitor group grew more slowly than those in the SW620-Vector group (Fig. 5i).

### Repression of GSK3β inhibits the CRC progression induced by miR-452

**Fig. 6:**
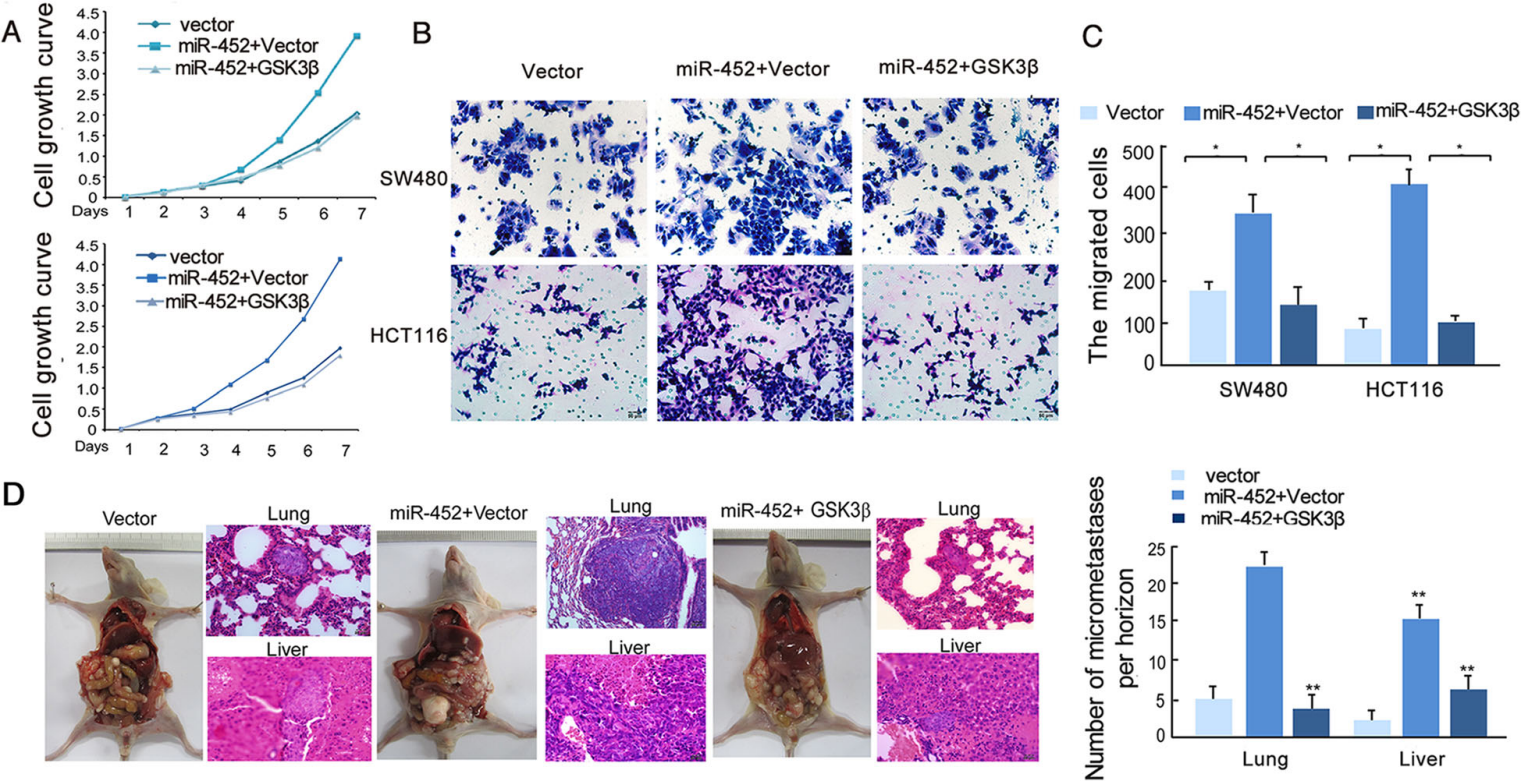
Function of miR-452 to promote CRC can be inbibited by repressing GSK3β. **a**, **b**, **c**, overexpression of GSK3β reversed cell growth induced by miR-452 as determined by MTT(A) and transwell assays (**b**, **c**). **d**, tumor metastasis model. Overexpression of GSK3β decreased metastasis induced by miR-452 (*n* = 6)

Furthermore, MTT (Fig. 6a) and transwell functional assays (Fig. 6b, c) were used to detect whether GSK3β can reverse, at least in part, the impact of miR-452 on sustained CRC cell proliferation and migration. The results show that exogenous overexpression of GSK3β inhibits the “oncomir” effect of miR-452 on CRC cells. To further determine the in vivo effects of GSK3β on diminishing the “oncomir” effect of miR-452 on CRC, SW480/miR452 cells or SW480/miR452 + GSK3β cells were orthotopically implanted into the cecum of nude mice (*n* = 6 for each group). As the results show, large primary tumors in the cecum and widespread distribution of tumor foci in the abdomen were observed in the SW480/miR452 group, whereas primary tumors in the SW480/miR452 + GSK3β group (Fig. 6d, Left) were remarkably smaller. Notably, metastatic loci were observed in the lungs and liver of mice implanted with miR452 overexpressing cells. No obvious micro-metastases were detected in SW480/miR452 + GSK3β mice (Fig. 6d).

### LEF1 associates with the promoter of miR-452 in CRC

**Fig. 7:**
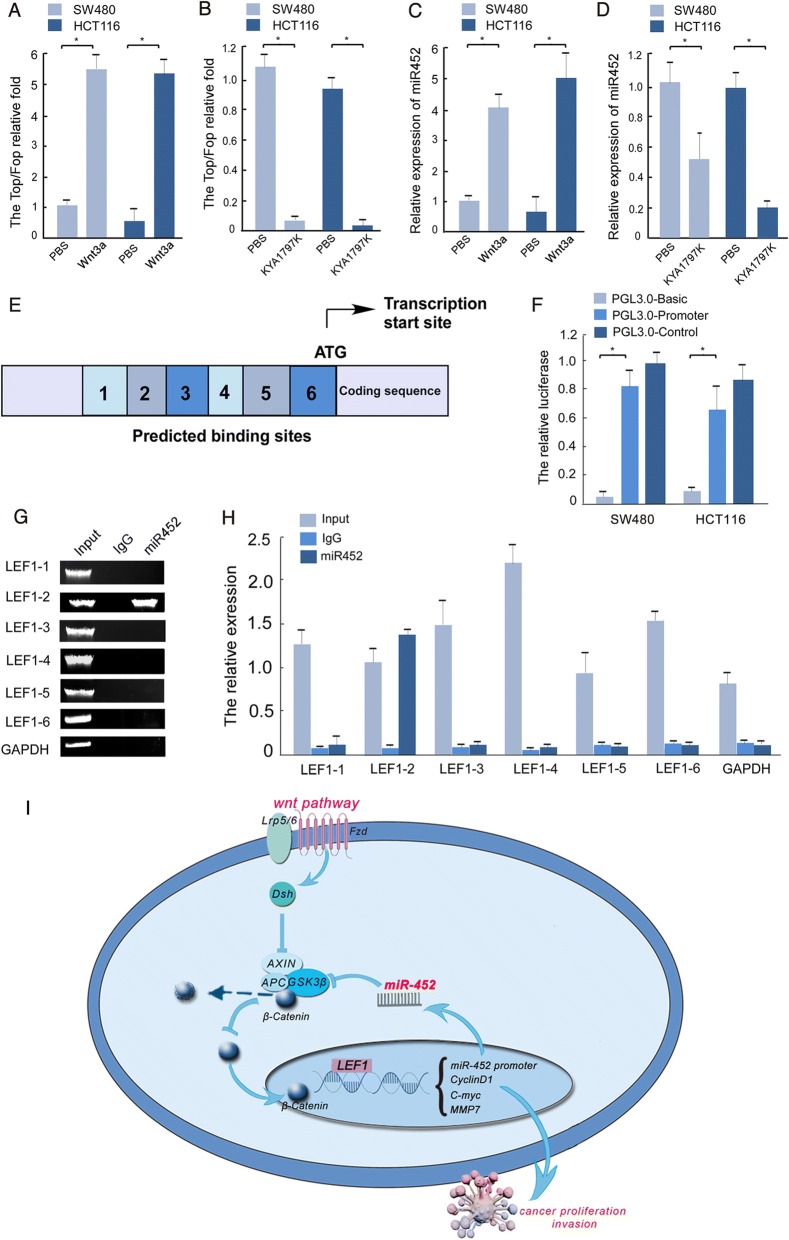
The promoter of miR-452 binds transcription factor LEF1. **a**, **b**, **c**, **d**, the results of top/fop and miR-452 expression in the indicated cells. The Wnt pathway was activated in cell lines treated with Wnt3a (**a**), and expression of miR-452 was upregulated in the same cell lines (**c**). The Wnt pathway was inhibited in cell lines treated with KYA1797K (**b**), and expression of miR-452 was downregulated in the same cell lines (**d**). **e**, the promoter of miR-452 contained 6 segments. **f**, luciferase assay was used to confirm the activity of the miR-452 promoter. **g**, **h**, the second segment of the miR-452 promoter binds to the LEF1 transcription factor based on PCR (**g**) and real-time PCR (**h**). **i**, MiR-452 promoted growth and invasion of colorectal cancer cell by regulating GSK3β-TCF4/LEF1 positive feedback loops

Two publicly available bioinformatic algorithms, PROMO and JASPAR, were used to predict the potential promoter of miR-452. The full-length miR-452 promoter region was subcloned into a luciferase vector and then a luciferase reporter assay was used to verify that the theoretical promoter region could modulate miR-452 transcription (Fig. 7f) in both SW480 and HCT116 cells. Using this reporter assay, increased miR-452 promoter activity was observed following Wnt signaling activation, suggesting that LEF1 could be the transcription factor responsible for miR-452 gene transcription. As shown in Fig. 7a, miR-452 promoter activity increased in the presence of Wnt3a, an activator of Wnt pathway signaling, and decreased in the presence of the Wnt signaling inhibitor KYA1797K (Fig. 7b). These results were consistently observed in SW480 and HCT116 cells compared to control cells treated with PBS. In addition, real-time PCR assays showed that Wnt3a increased miR-452 expression (Fig. 7c) while KYA1797K decreased miR-452 expression (Fig. 7d).

As shown in Fig. 7e, the promoter of miR-452 was separated into six fragments. ChIP was performed using an anti-LEF1 antibody or a control immunoglobulin G to identify LEF1 binding sites in the miR-452 promoter in SW480 cells. The p120 and GAPDH promoters were used as positive and negative controls, respectively. As shown in Fig. 7g and h, the second fragment has high binding affinity for LEF1. We recycled the DNA and utilized real-time PCR to verify that the second region of the miR-452 promoter could be pulled down by the anti-LEF1 antibody. Taken together, these results identified LEF1 as a direct transcriptional target of miR-452.

## Discussion

In this study, we produced evidence regarding the role of miR-452 in CRC initiation and progression. MiR-452 was found to have a direct role in promoting Wnt/β-catenin signaling through binding the 3’-UTR of GSK3β. We further demonstrated that miR-452 can be induced by the crucial downstream transcription factor TCF4/LEF1, which is activated by Wnt signaling.

MiRNAs are a group of small regulatory non-coding RNA molecules that suppress target gene expression and protein translation via targeting mRNA specific 3’-UTRs. Accumulating evidence suggests that miRNAs are of great importance in tumor development and recent studies have shown that numerous miRNAs are deregulated in CRC [22, 23], and correlate with CRC pathological stage and prognosis. It has been reported that miR-452 is a member of the miR-224/miR-452 cluster [15, 24]. Our previous studies validated that miR-224 deregulation is essential for CRC occurrence and development [9, 10]. A study by Hui Ling et al. reported the clinical and biological significance of miR-224 expression in CRC metastasis [25]. MiR-224 clearly acts as an oncogene and plays a major role in sustaining an aggressive CRC phenotype. Helle Kristensen et al. found that miR-452 cooperates with miR-224 and GABRE and that miR-452 expression predicts biochemical recurrence after radical prostatectomy in prostate cancer [15]. Altered miR-452 expression has been reported in several malignancies, yet little is known about miR-452 in CRC. To explore the role of miR-452 in CRC, we first examined miR-452 expression in 43 matched pairs of samples from CRC patients and found that miR-452 was expressed dramatically higher in human CRC tissues compared to para-carcinoma normal tissues. We further explored the relationship between miR-452 expression and clinicopathological features in CRC and found that upregulation of miR-452 expression was positively associated with T classification. In addition, Kaplan–Meier test implied that high miR-452 expression significantly correlated with CRC severity.

Next, we attempted to determine the potential molecular mechanism by which miR-452 sustains the aggressive clinical phenotype of CRC. One of the most remarkable findings in recent years has been that the vast majority of CRC patients carry mutations in one of two genes involved in the canonical Wnt/β-catenin signaling pathway [26–28], namely, the APC and β-catenin (CTNNB1) [29] genes. More specifically, it has been reported that approximately 90% of CRC tumors have mutations in either APC or β-catenin (CTNNB1) [29]. Moreover, mutation of either the APC or CTNNB1 gene generally results in cytoplasmic accumulation of β-catenin protein, which subsequently induces multistep colorectal carcinogenesis. Obviously, continuous activation of Wnt signaling is crucial for CRC initiation and progression. In addition, our previous study showed that miR-224 plays a crucial role in aberrant Wnt signaling during CRC initiation and progression [10]. This prompted us to investigate whether modulation of the aggressive phenotype of CRC by miR-452 was at least, in part, due to regulation of Wnt signaling. Gene database analysis showed that GSK3β, a negative regulator of Wnt signaling, was a potential miR-452 target gene. A luciferase assay validated that miR-452 directly interacts with the 3’-UTR of GSK3β. Recent evidence indicates that GSK3β acts as a tumor suppressor gene and that its expression is continually depleted in many human cancers including breast cancer, prostate cancer, and colorectal cancer. In addition, our data demonstrated that miR-452 activates Wnt signaling and subsequently recruits β-catenin to the nucleus. As a result of β-catenin nuclear accumulation, direct or indirect downstream molecules of Wnt signaling, including c-Myc [30, 31], cyclinD1 and MMP7, are modulated by miR-452.

The β-catenin protein relocates to nucleus where it acts as a coactivator for the TCF/LEF family of transcription factors [32]. TCF/LEF transcription factors transactivate a wide variety of target genes including JUN, MYC, cyclinD1, MDR1, MMP7, and AXIN2, as well as many other microRNAs that are involved in the invasion, differentiation, proliferation, and apoptosis of intestinal epithelial cells [33]. Furthermore, we demonstrated that exogenous overexpression of miR-452 facilitated CRC cell growth in vitro and in vivo. Increased cellular migration and invasion were also observed. These results are clearly consistent with previous studies demonstrating that miR-452 might act as an oncomir for CRC progression. The observation that in a way CRC progression can be attributed to up-regulate miR-452 when Wnt signaling was activated, is noteworthy. This prompted us to explore the mechanism behind this result. We used an online website to predict the promoter of miR-452 and were amazed to find that LEF1, a member of the TCF/LEF transcription factor family, potentially acts as a transcription factor that binds to the upstream promoter of miR-452. Finally, we validated this hypothesis by performing a ChIP assay and further experiments (Fig. 7i).

Accumulating evidences demonstrated that a specific miRNA had diverse roles in different cancers [34]. Zheng et al. found SOX7 was the target gene of miR-452, which could bind with β-catenin and TCF4 in the nucleus and then inhibit the activity of Wnt/β-catenin signaling pathway [18]. Furthermore, miR-452 promoted stem-like traits in hepatocellular carcinoma [18]. As we known, Wnt/β-catenin signaling was reported to exert crucial roles in the maintenance of self-renewal for stem cells [35]. We found miR-452 could activate Wnt/β-catenin signaling pathway by targeting GSK3β in colorectal cancer. Therefore, the cosslink between miR-452 and stem cells in colorectal cancer still needs us to explore.

In summary, a miR-452-GSK3β-TCF4/LEF1 positive feedback loop plays a pivotal role in regulating CRC initiation and progression. Functional experiments demonstrate that miR-452 acts as a stimulator of Wnt signaling and subsequently induces CRC cell proliferation, invasion and metastasis by directly targeting GSK3β. In turn, the direct downstream transcription factor LEF1 stimulates transcription of miR-452. The miR-452-GSK3β-TCF4/LEF1 positive feedback loop might offer a promising therapeutic target for CRC treatment.

## Conclusions

In summary, our results highlight an important role for miR-452 in the regulation of proliferation and migration in the pathogenesis of CRC by miR-452-GSK3β-TCF4/LEF1 positive feedback loop, and miR-452 could be considered as a potential prognostic marker and/or as an effective therapeutic target for CRC.

## Additional files

Additional file 1: **Figure S2.** Primer sequenced used for quantitative real-time PCR. (TIF 326 kb)

Additional file 2: Supplemental files. (ZIP 13000 kb)

Additional file 3: **Figure S3.** Chip primer sequenced used for quantitative real-time PCR. (TIF 319 kb)

Additional file 4: **Figure S1.** MiR-452 expression in CRC cells and transfection efficency of miR-452/GSK3β. (A) The relative exprssion of miR-452 in 10 colorectal cancer cells by qPCR. (B) Western blot analyses of GSKβ in cell transfected with Vector, miR-452, miR-452/GSK3β in SW480 and HCT116 cells. (C) Western blot analyses of β-catenin in the indicated cells. (D) Real-time quantitative PCR analysis of mRNA relative expression of β-catenin in the indicated cells. (TIF 14145 kb)

## Abbreviations

CRC: Colorectal cancer
TCF4/LEF1: T-cell factor4/lymphoid enhancer factor1
