# Supplementary figures and images for: MiR-452 promotes an aggressive colorectal cancer phenotype by regulating a Wnt/β-catenin positive feedback loop
Source: J Exp Clin Cancer Res. 2018 Sep 25;37:238. doi: 10.1186/s13046-018-0879-z (PMC6156870; doi:10.1186/s13046-018-0879-z)

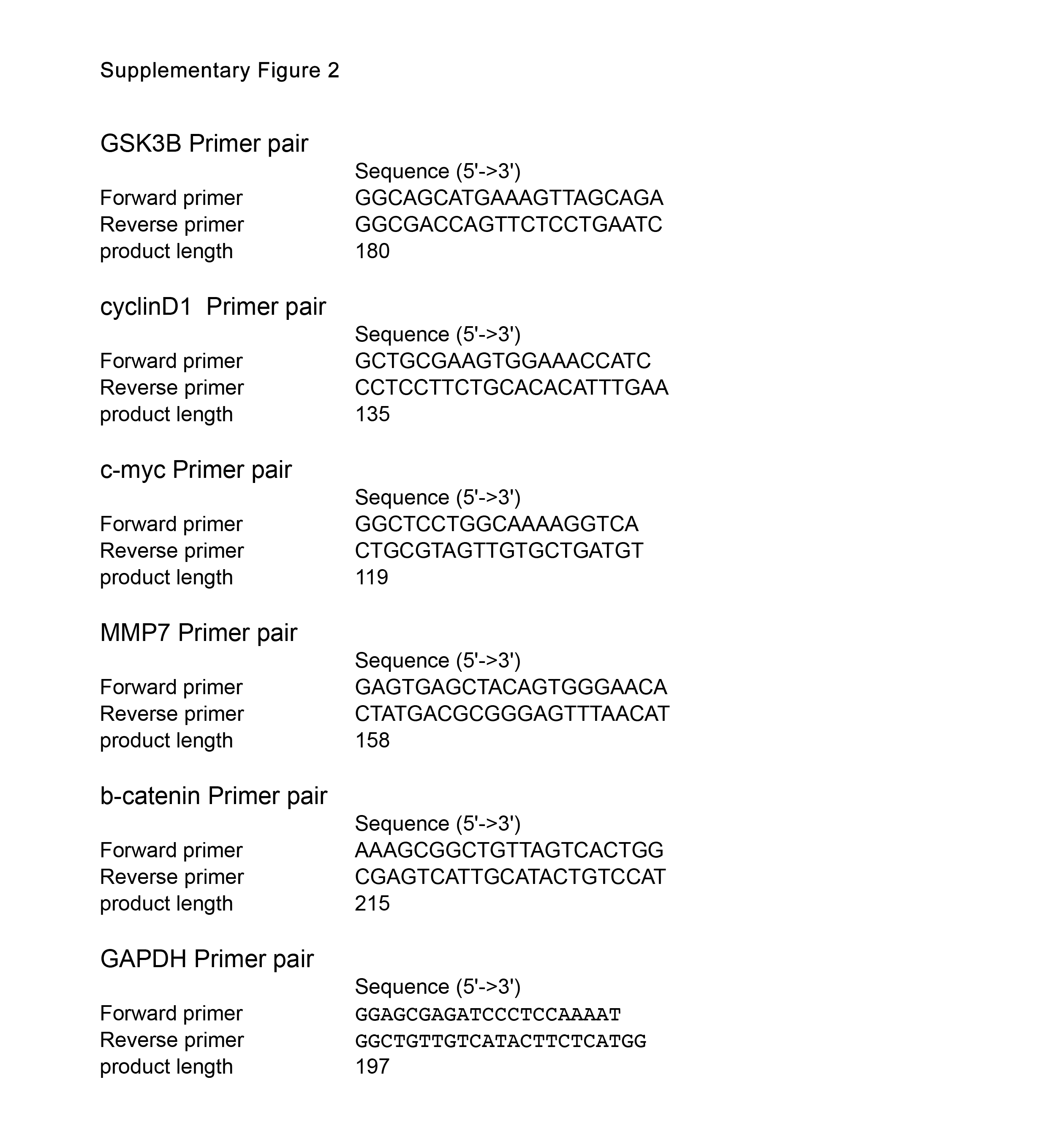

Supplement: Supplementary file 1 — Figure S2. Primer sequenced used for quantitative real-time PCR. (TIF 326 kb) [file 13046_2018_879_MOESM1_ESM.tif]

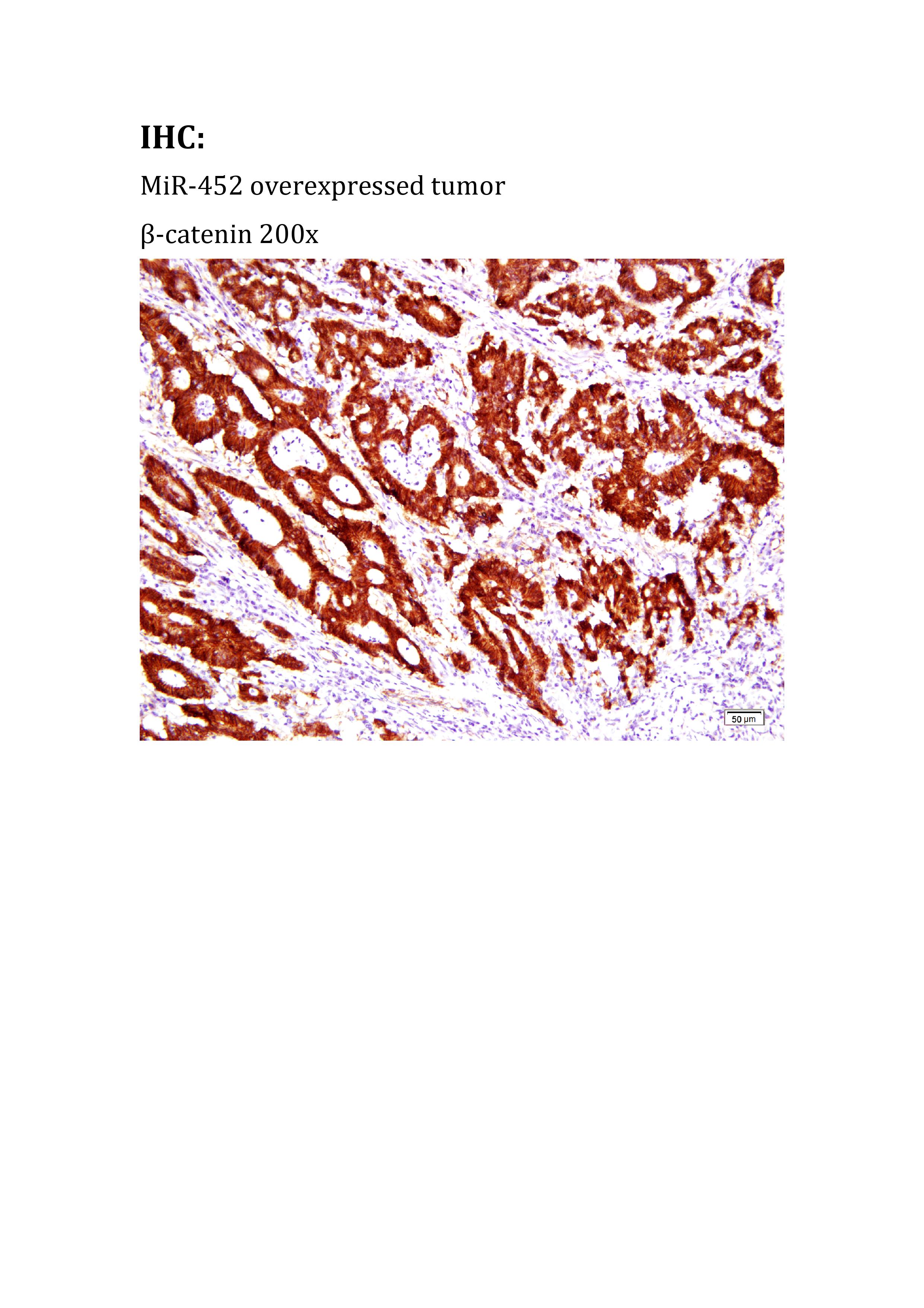

Supplement: Supplementary file 2 — Supplemental files. (ZIP 13000 kb) [file 13046_2018_879_MOESM2_ESM.zip › 0001.jpg]

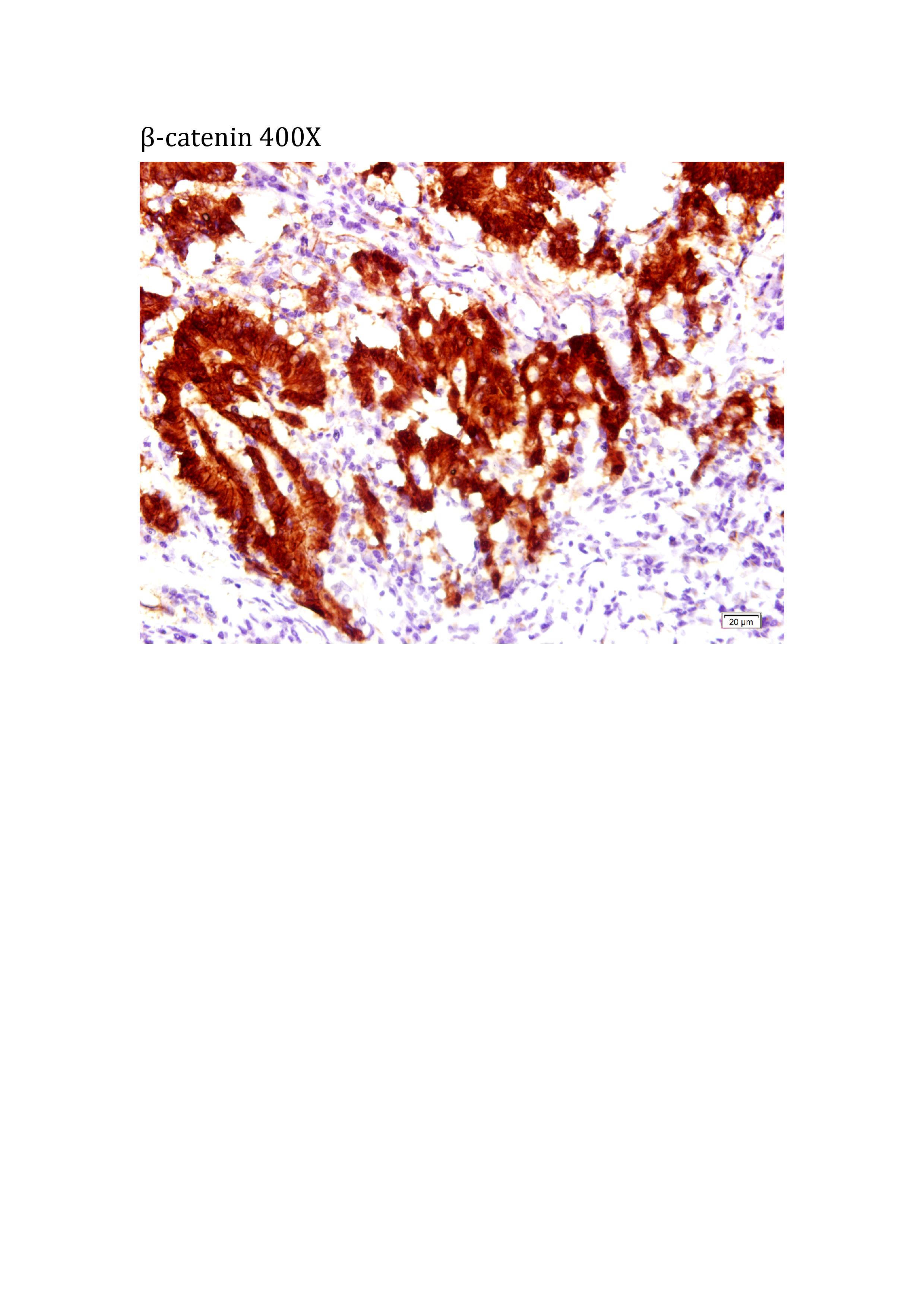

Supplement: Supplementary file 2 — Supplemental files. (ZIP 13000 kb) [file 13046_2018_879_MOESM2_ESM.zip › 0002.jpg]

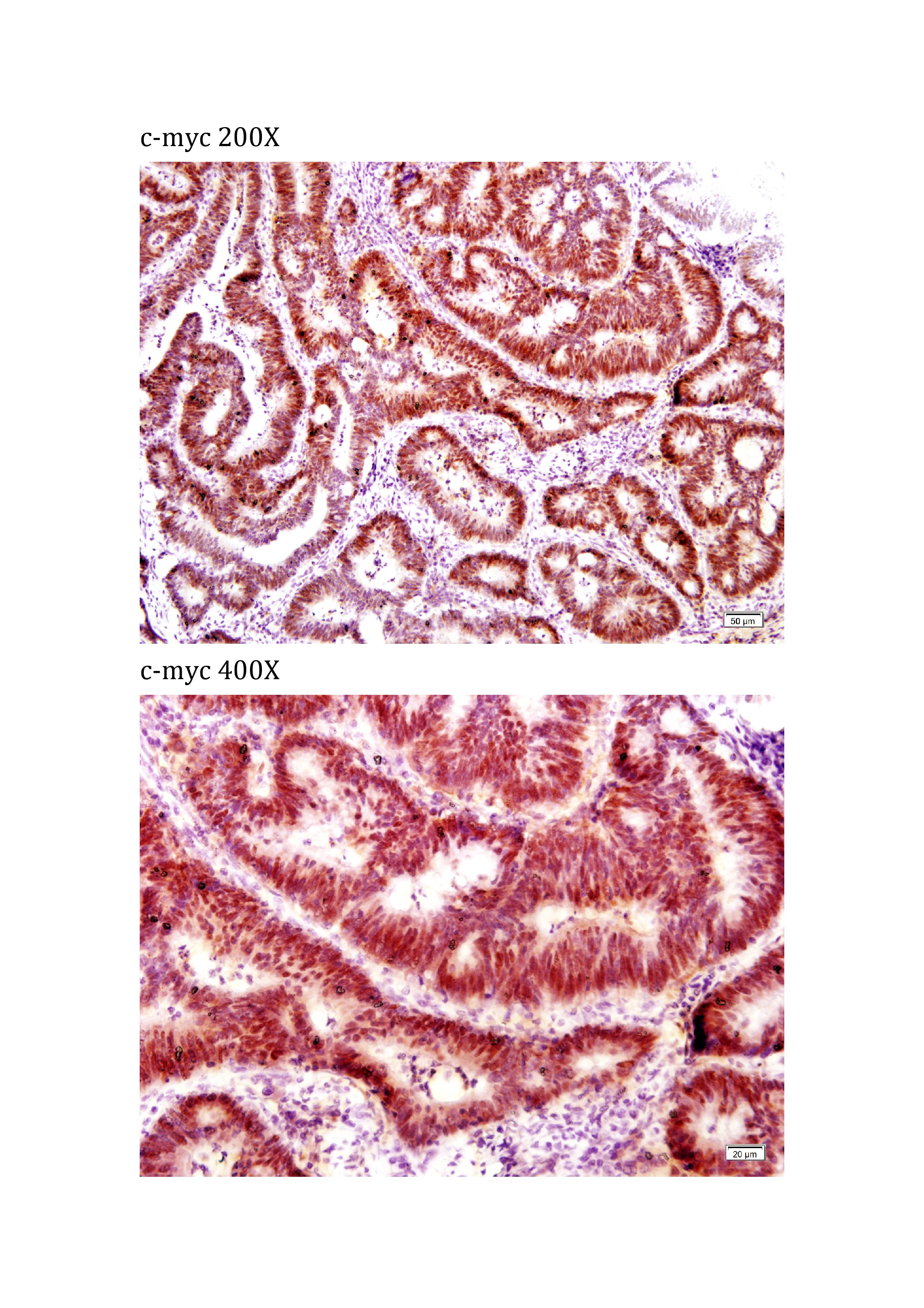

Supplement: Supplementary file 2 — Supplemental files. (ZIP 13000 kb) [file 13046_2018_879_MOESM2_ESM.zip › 0003.jpg]

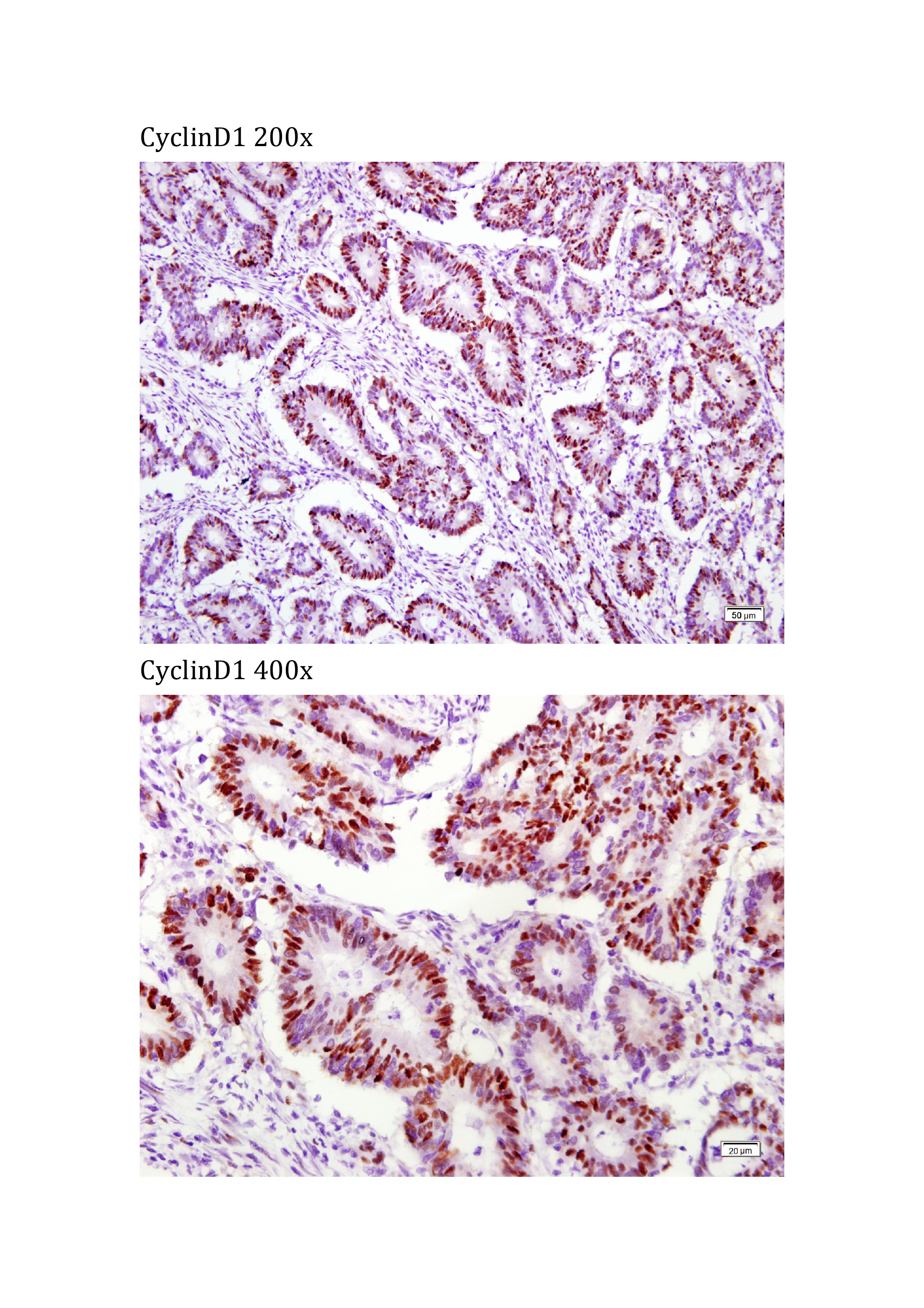

Supplement: Supplementary file 2 — Supplemental files. (ZIP 13000 kb) [file 13046_2018_879_MOESM2_ESM.zip › 0004.jpg]

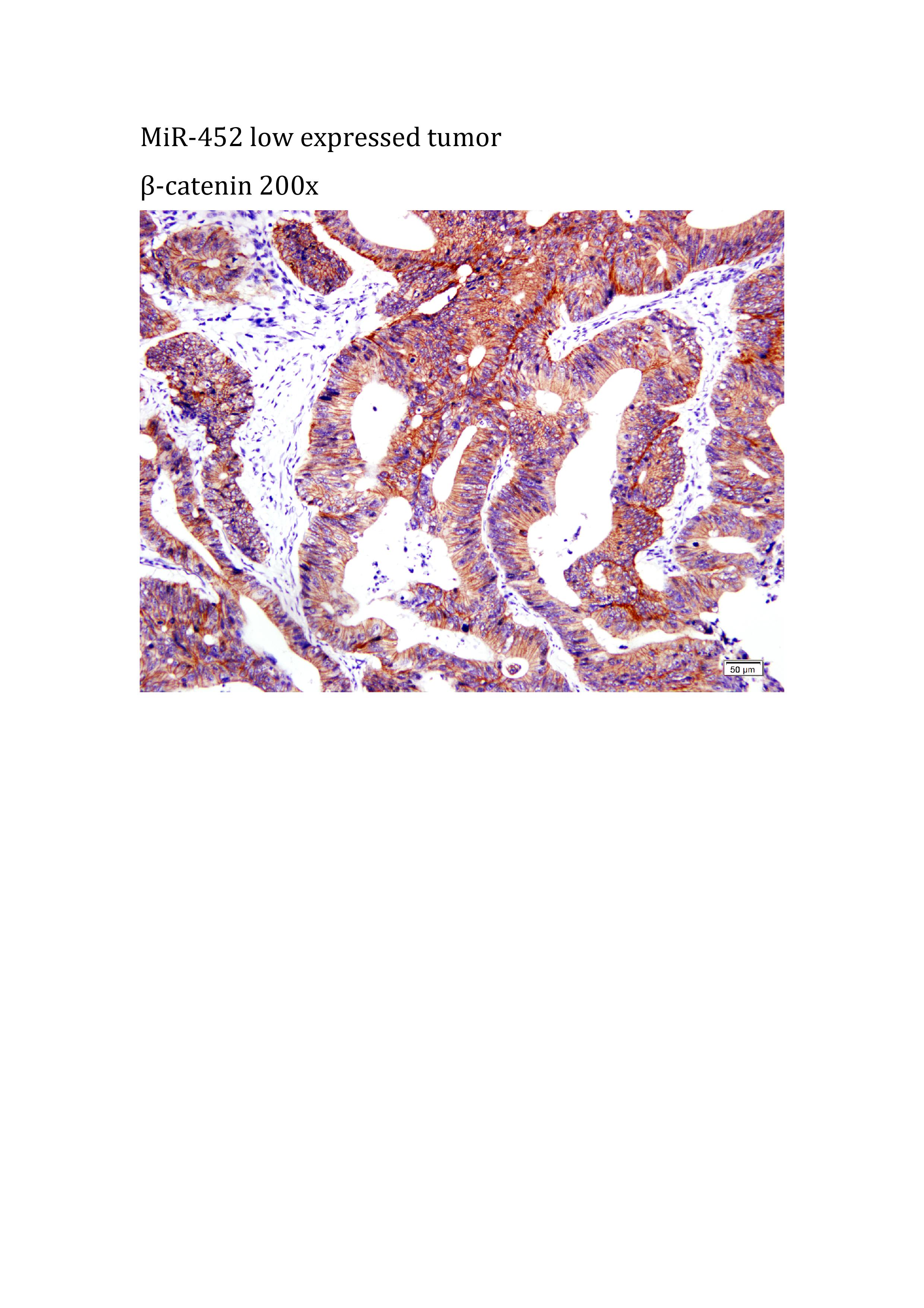

Supplement: Supplementary file 2 — Supplemental files. (ZIP 13000 kb) [file 13046_2018_879_MOESM2_ESM.zip › 0005.jpg]

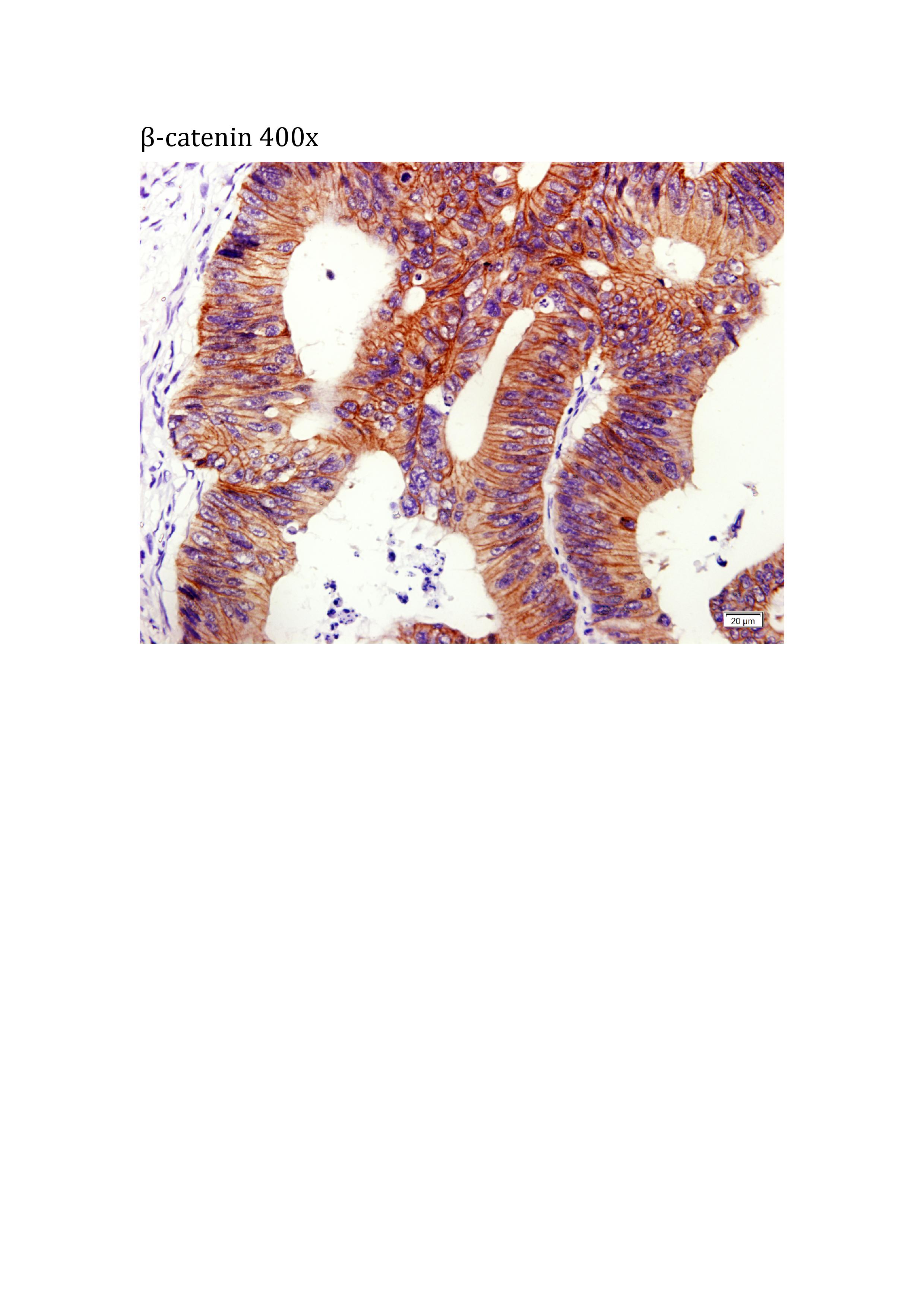

Supplement: Supplementary file 2 — Supplemental files. (ZIP 13000 kb) [file 13046_2018_879_MOESM2_ESM.zip › 0006.jpg]

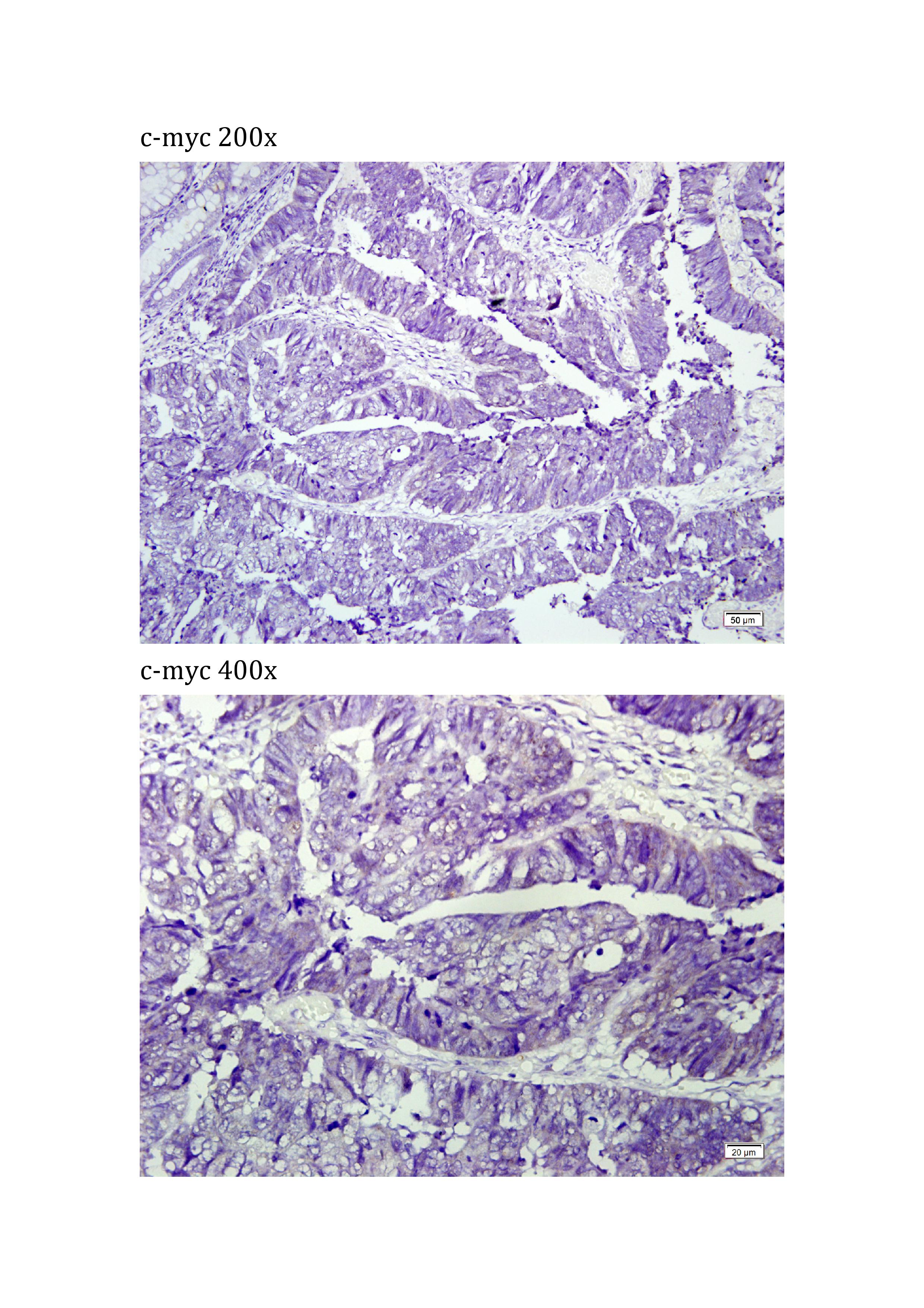

Supplement: Supplementary file 2 — Supplemental files. (ZIP 13000 kb) [file 13046_2018_879_MOESM2_ESM.zip › 0007.jpg]

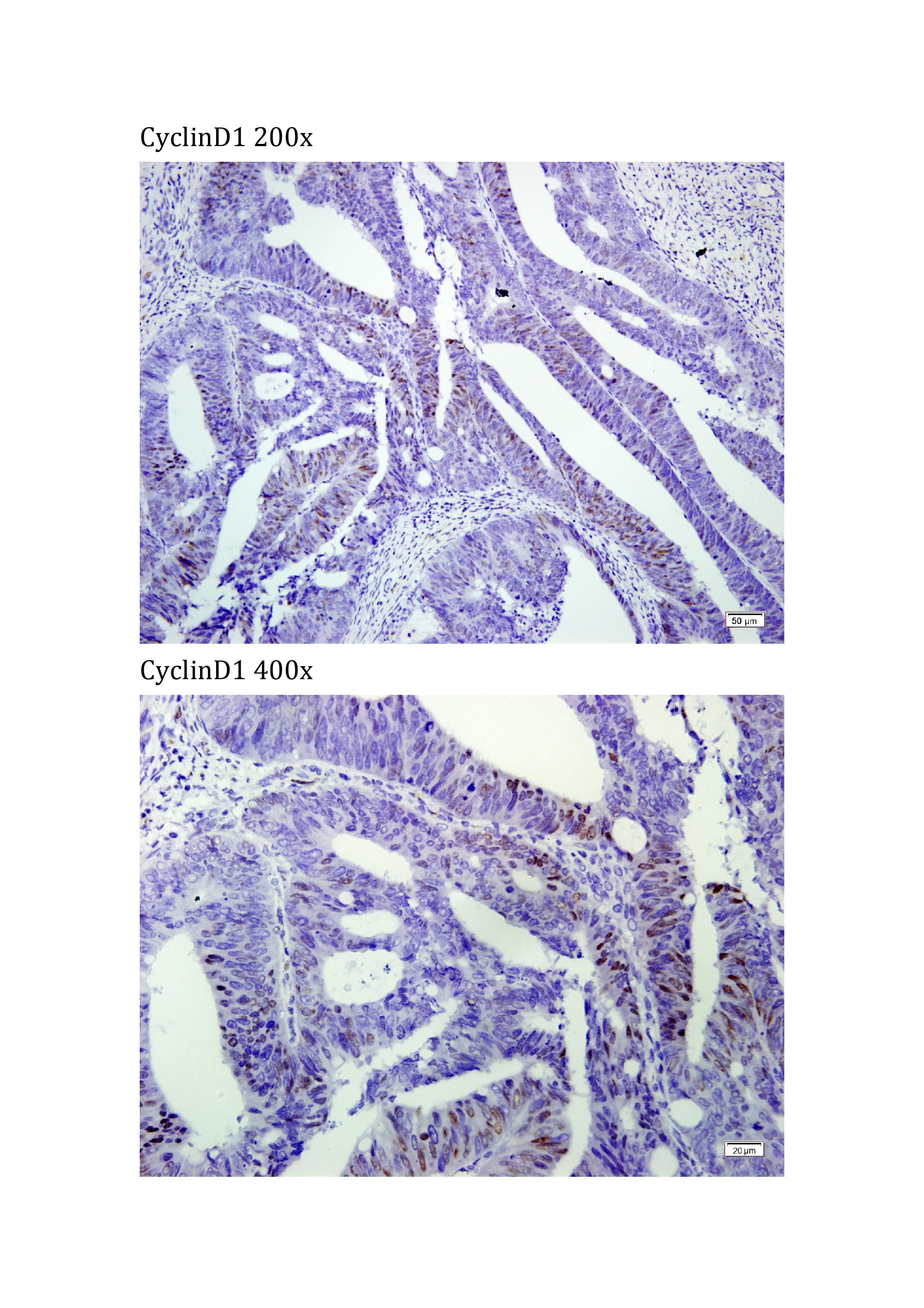

Supplement: Supplementary file 2 — Supplemental files. (ZIP 13000 kb) [file 13046_2018_879_MOESM2_ESM.zip › 0008.jpg]

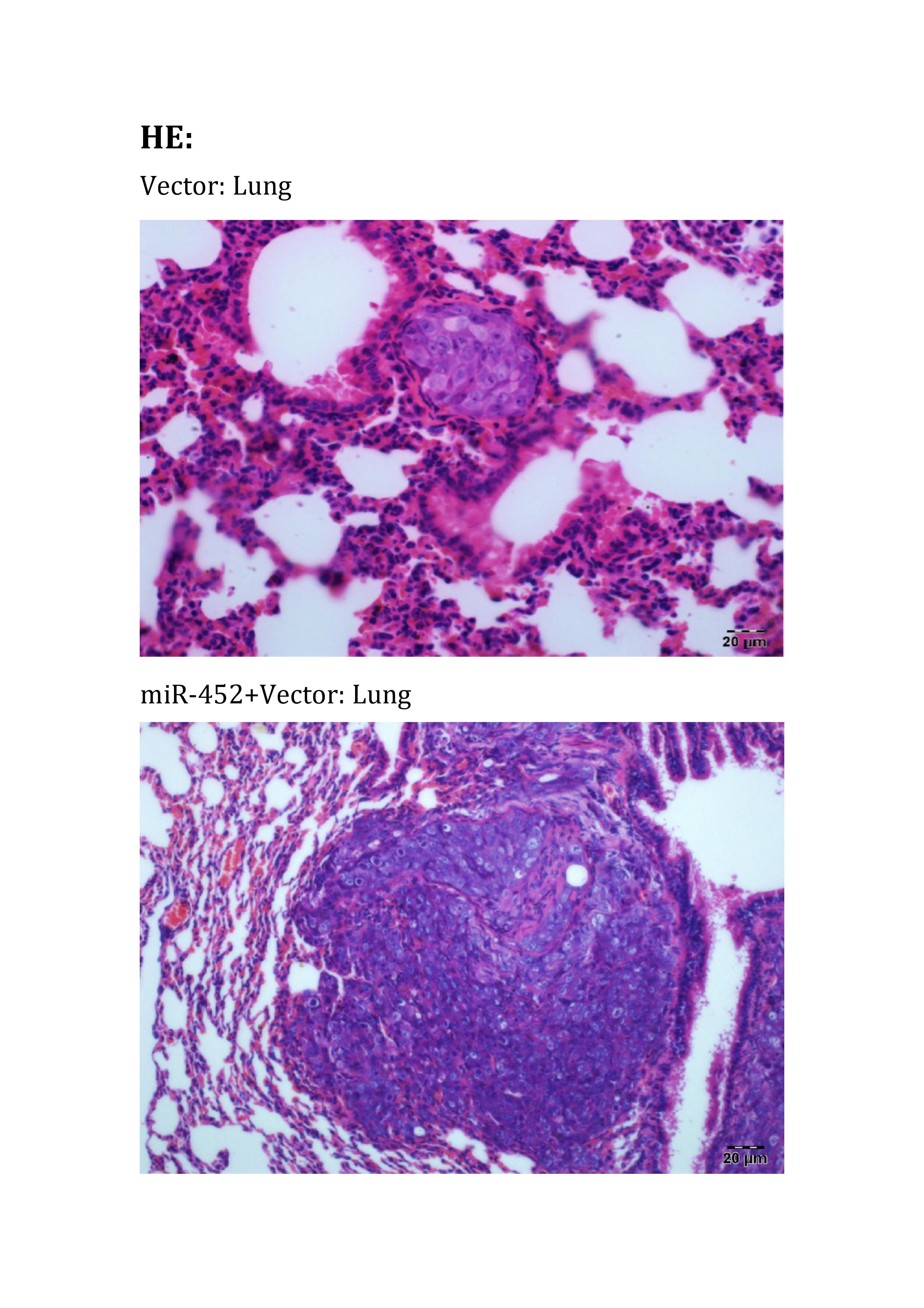

Supplement: Supplementary file 2 — Supplemental files. (ZIP 13000 kb) [file 13046_2018_879_MOESM2_ESM.zip › 0009.jpg]

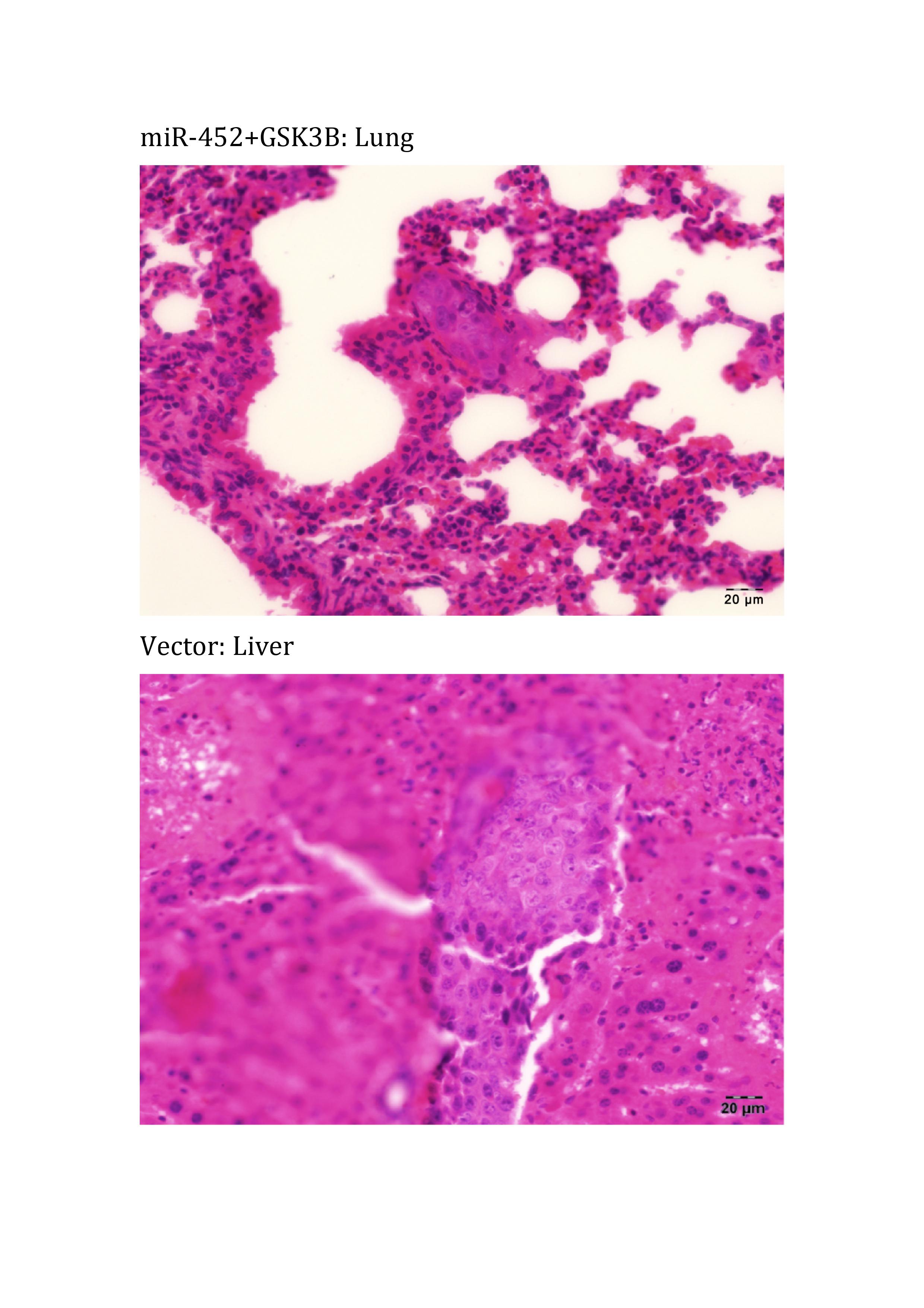

Supplement: Supplementary file 2 — Supplemental files. (ZIP 13000 kb) [file 13046_2018_879_MOESM2_ESM.zip › 0010.jpg]

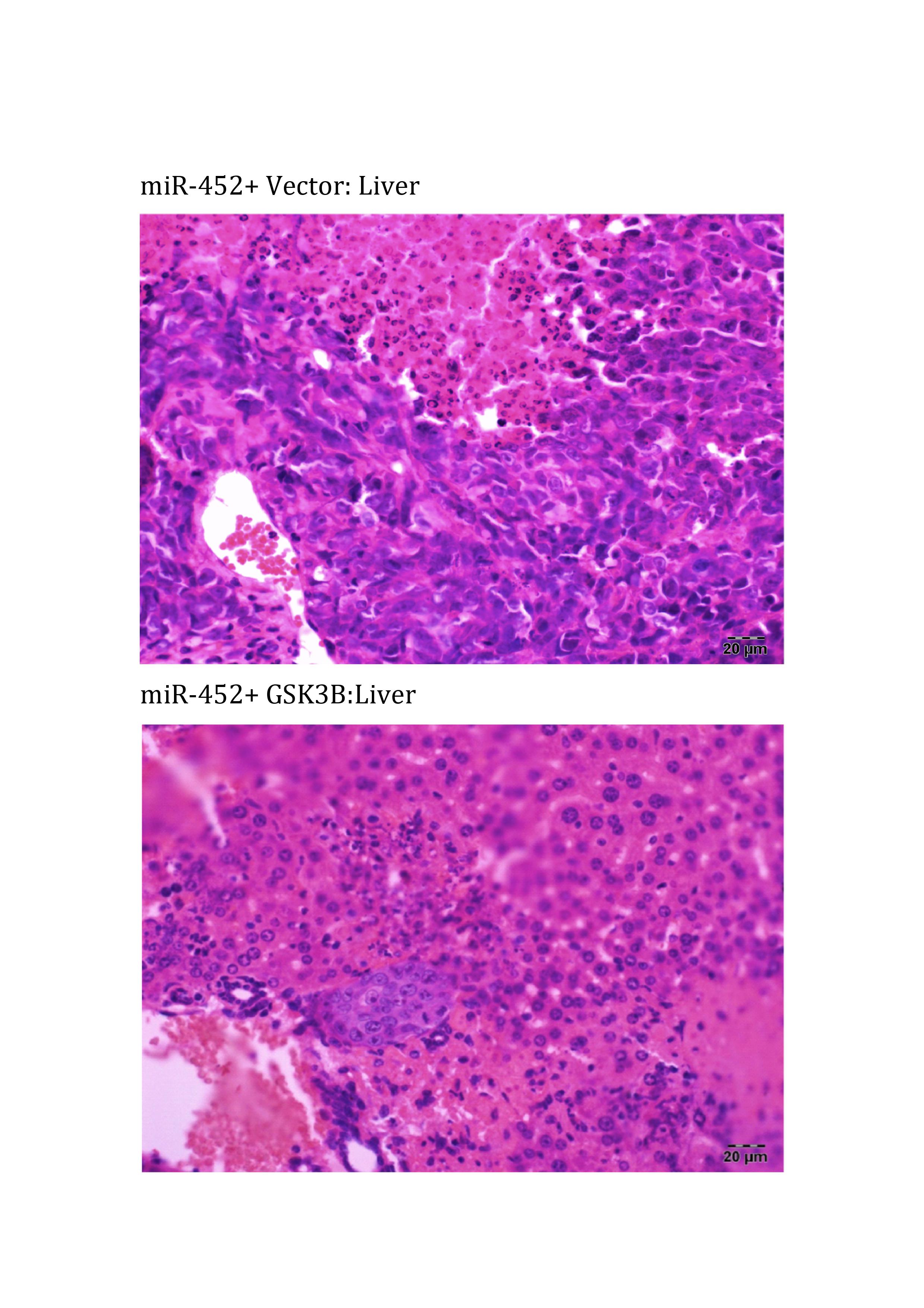

Supplement: Supplementary file 2 — Supplemental files. (ZIP 13000 kb) [file 13046_2018_879_MOESM2_ESM.zip › 0011.jpg]

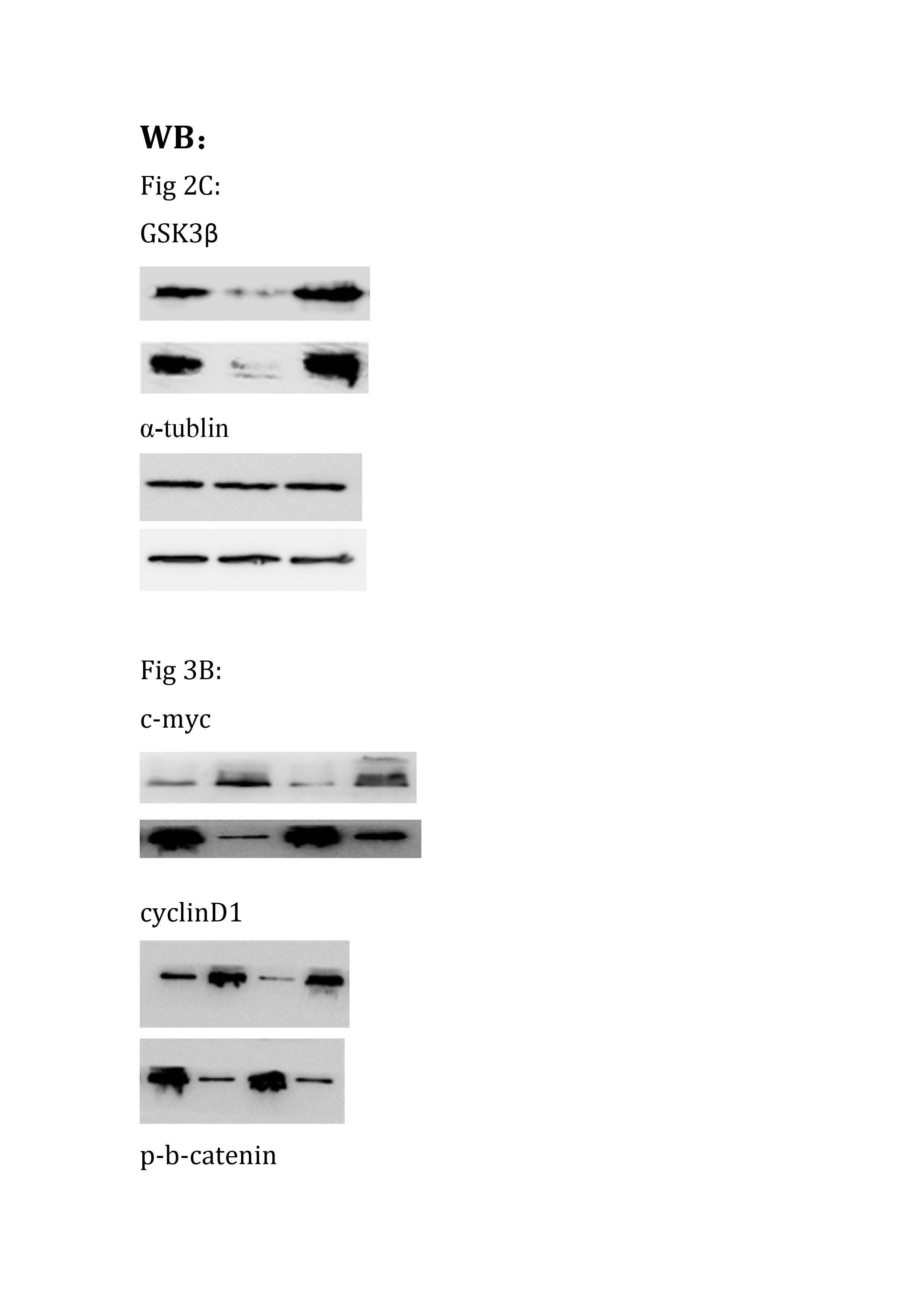

Supplement: Supplementary file 2 — Supplemental files. (ZIP 13000 kb) [file 13046_2018_879_MOESM2_ESM.zip › 0012.jpg]

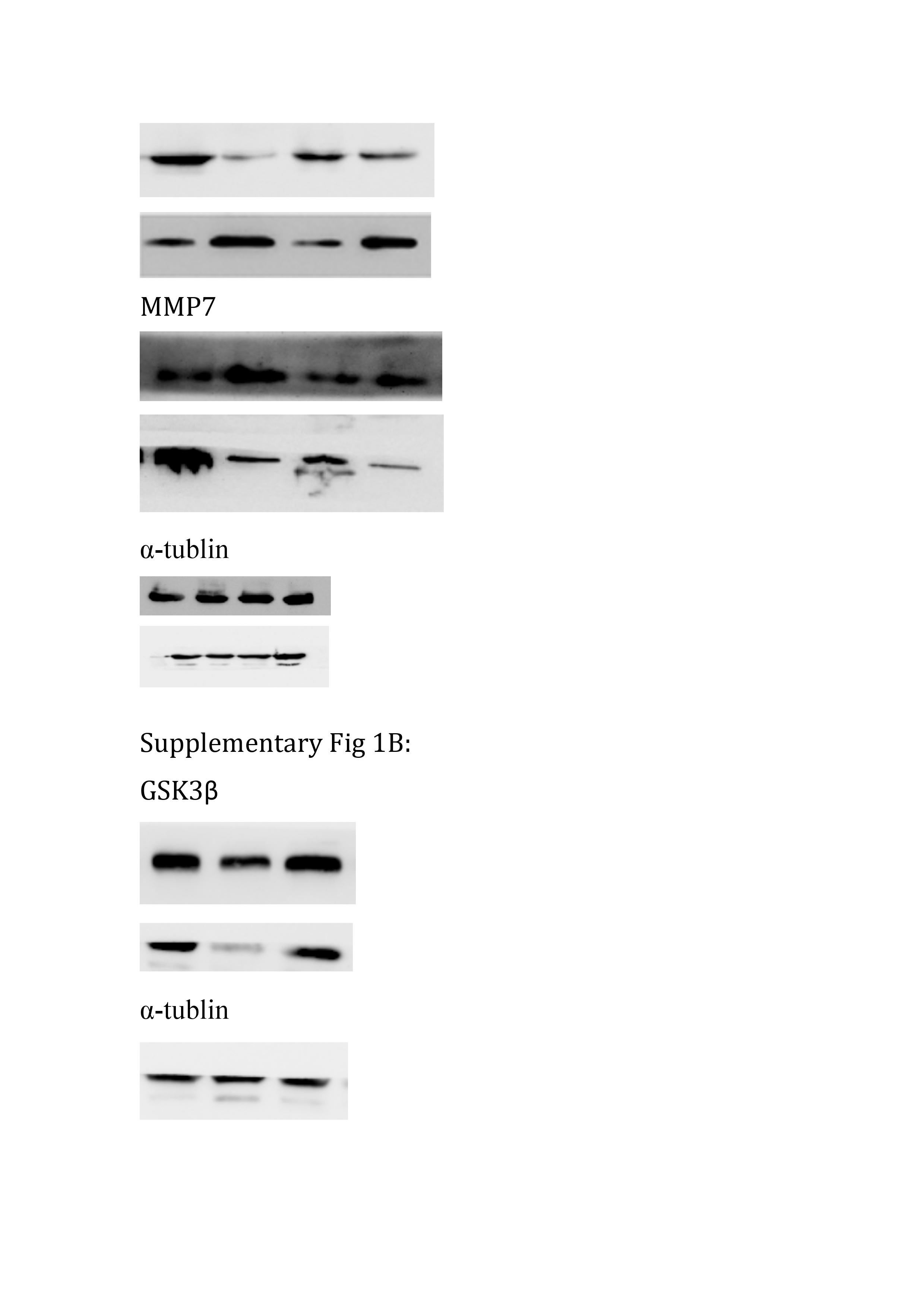

Supplement: Supplementary file 2 — Supplemental files. (ZIP 13000 kb) [file 13046_2018_879_MOESM2_ESM.zip › 0013.jpg]

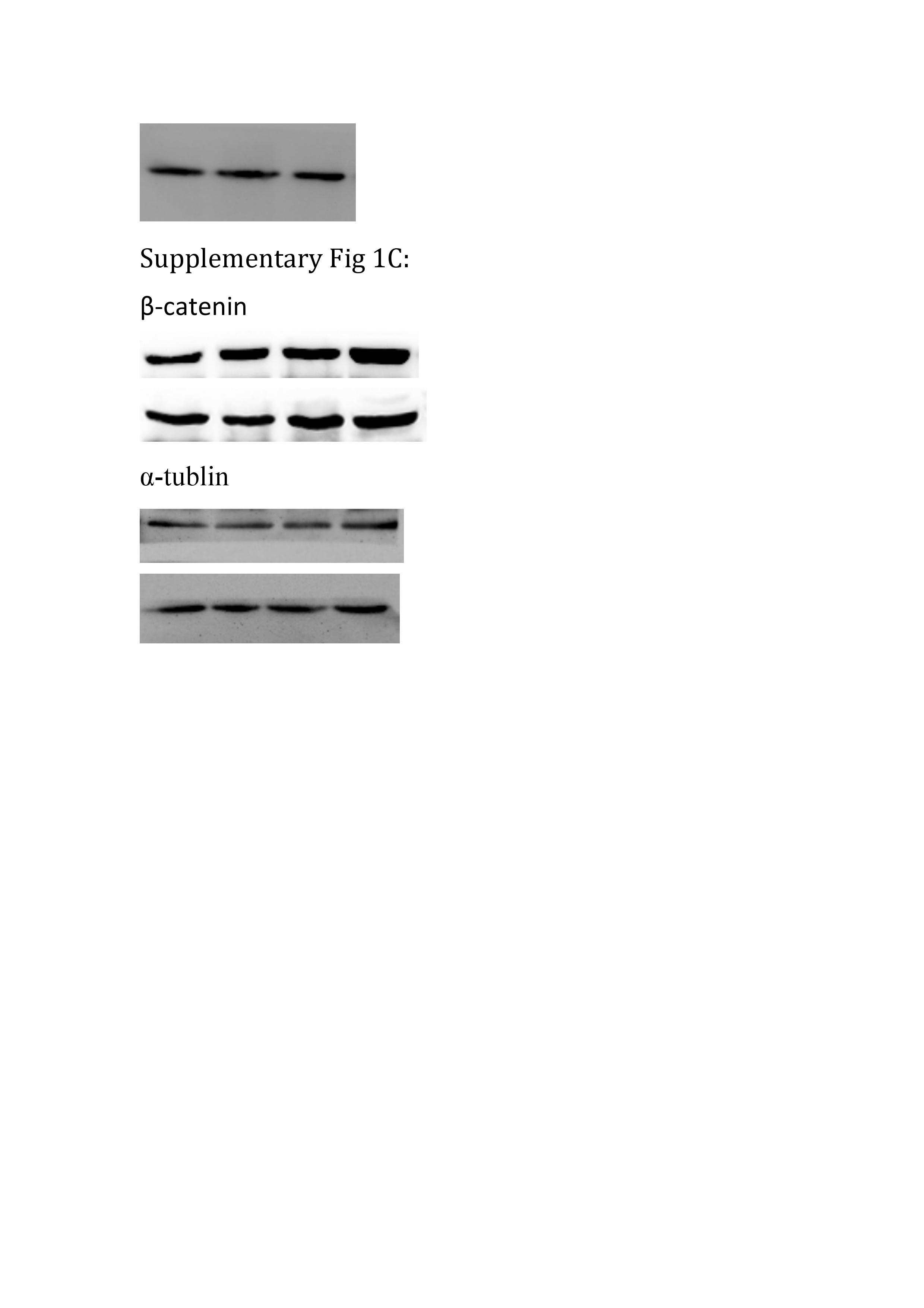

Supplement: Supplementary file 2 — Supplemental files. (ZIP 13000 kb) [file 13046_2018_879_MOESM2_ESM.zip › 0014.jpg]

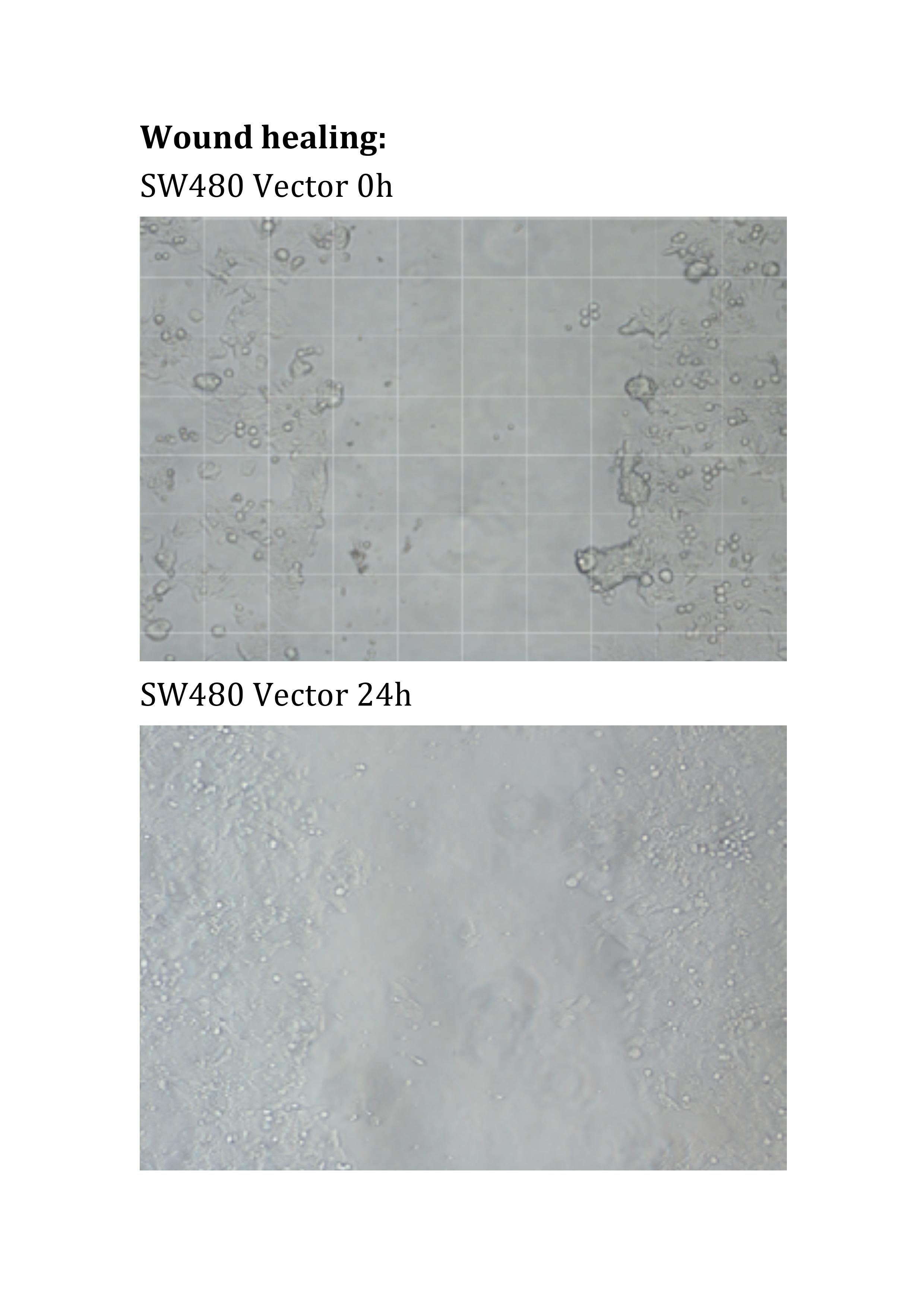

Supplement: Supplementary file 2 — Supplemental files. (ZIP 13000 kb) [file 13046_2018_879_MOESM2_ESM.zip › 0015.jpg]

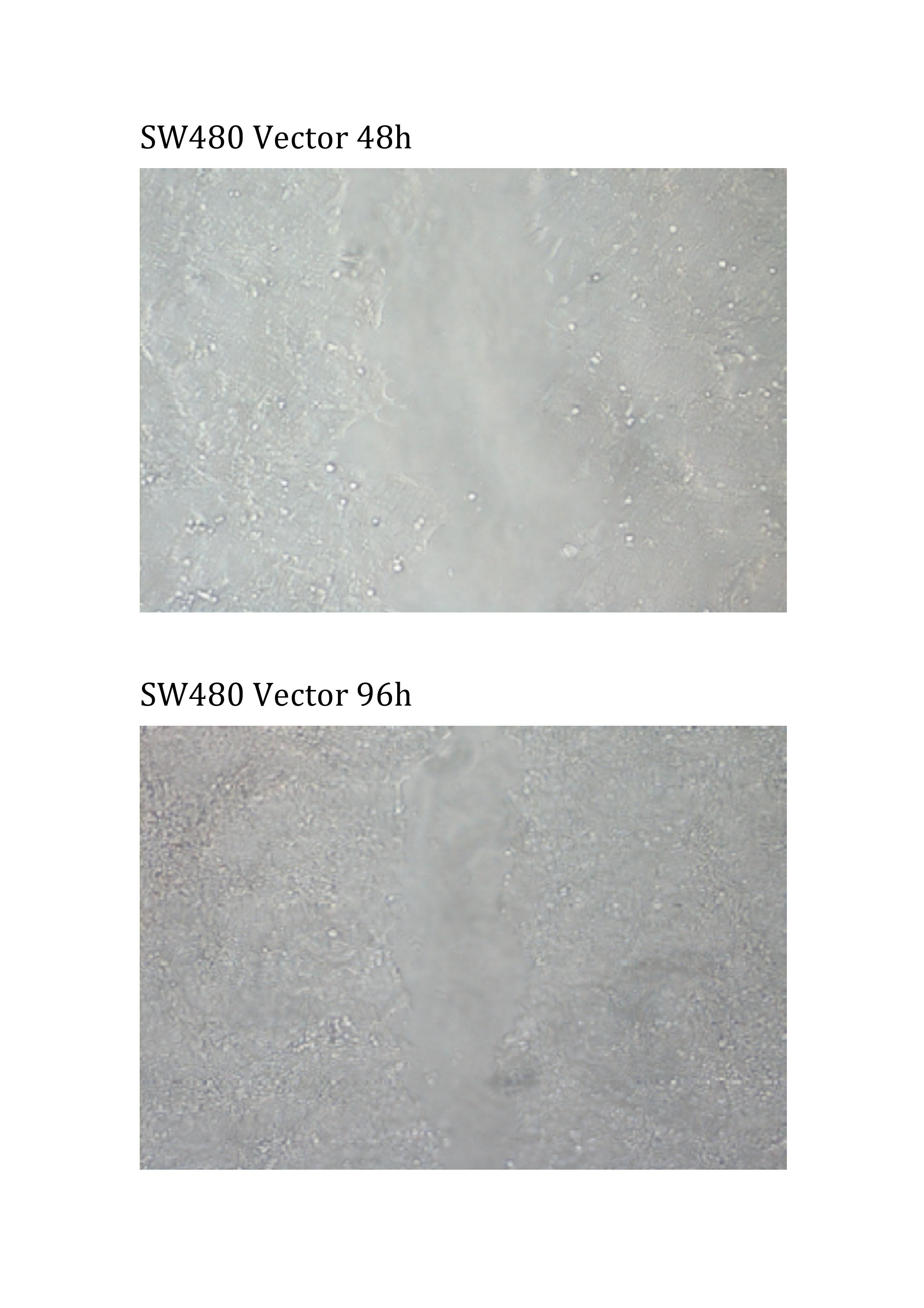

Supplement: Supplementary file 2 — Supplemental files. (ZIP 13000 kb) [file 13046_2018_879_MOESM2_ESM.zip › 0016.jpg]

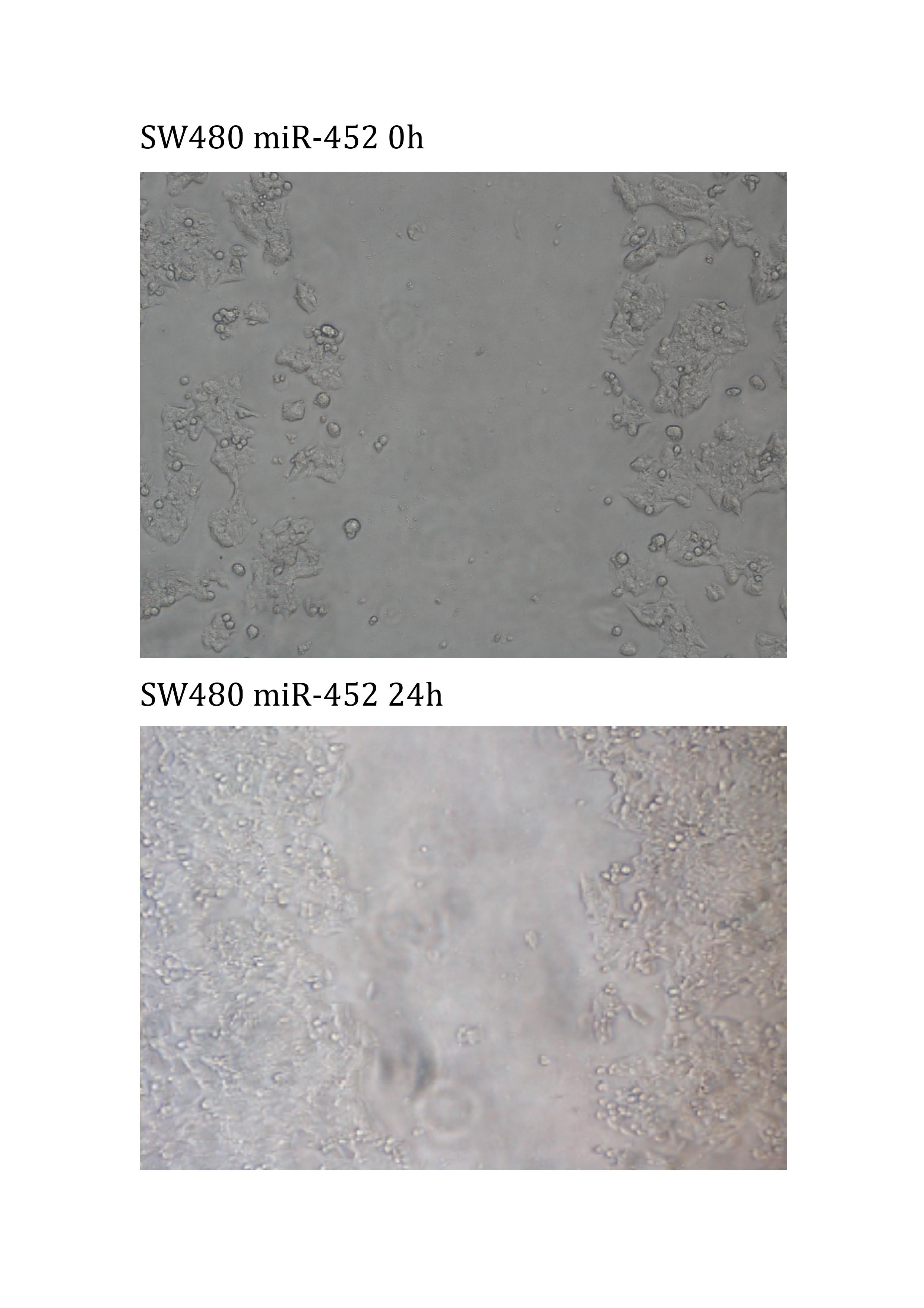

Supplement: Supplementary file 2 — Supplemental files. (ZIP 13000 kb) [file 13046_2018_879_MOESM2_ESM.zip › 0017.jpg]

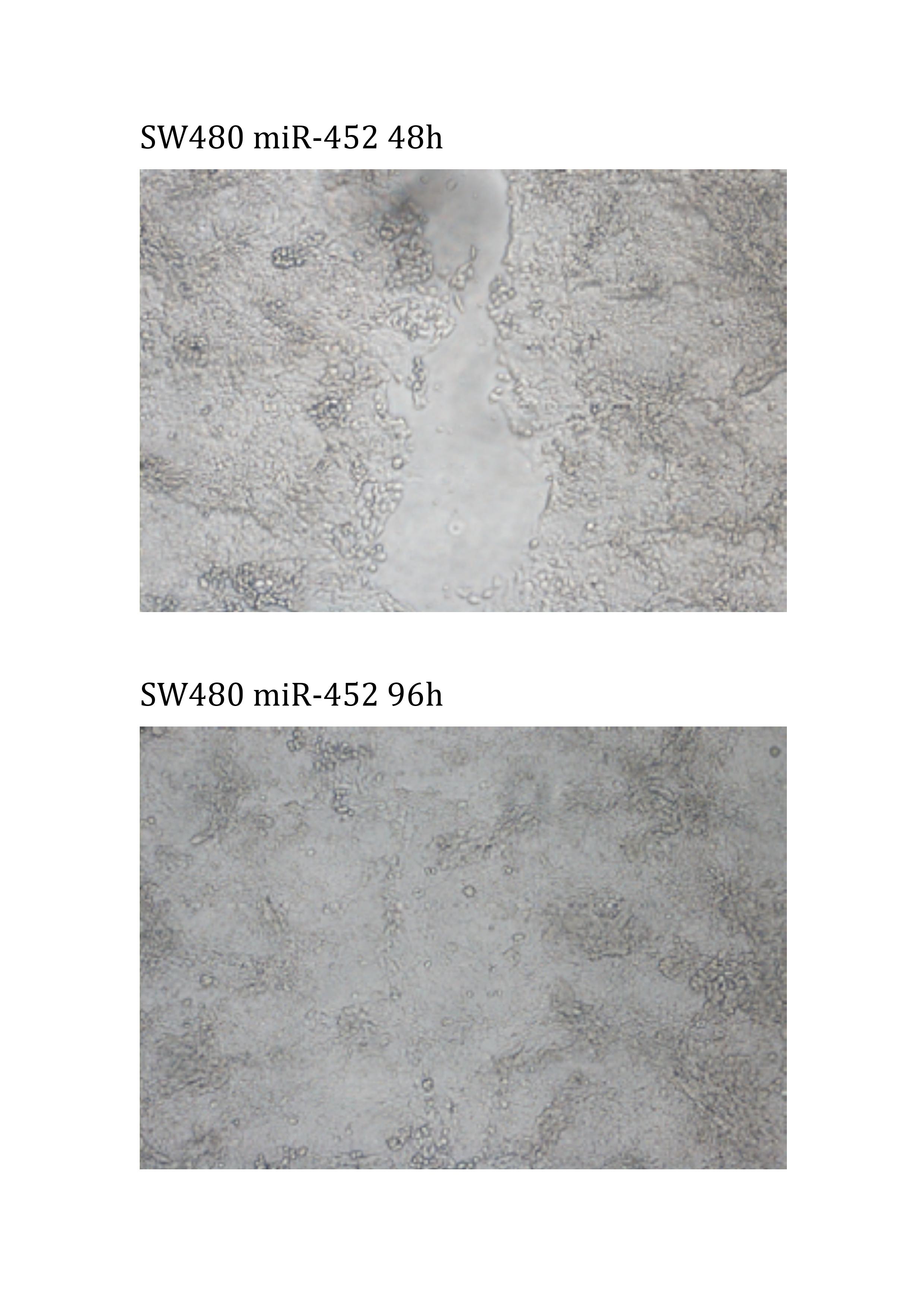

Supplement: Supplementary file 2 — Supplemental files. (ZIP 13000 kb) [file 13046_2018_879_MOESM2_ESM.zip › 0018.jpg]

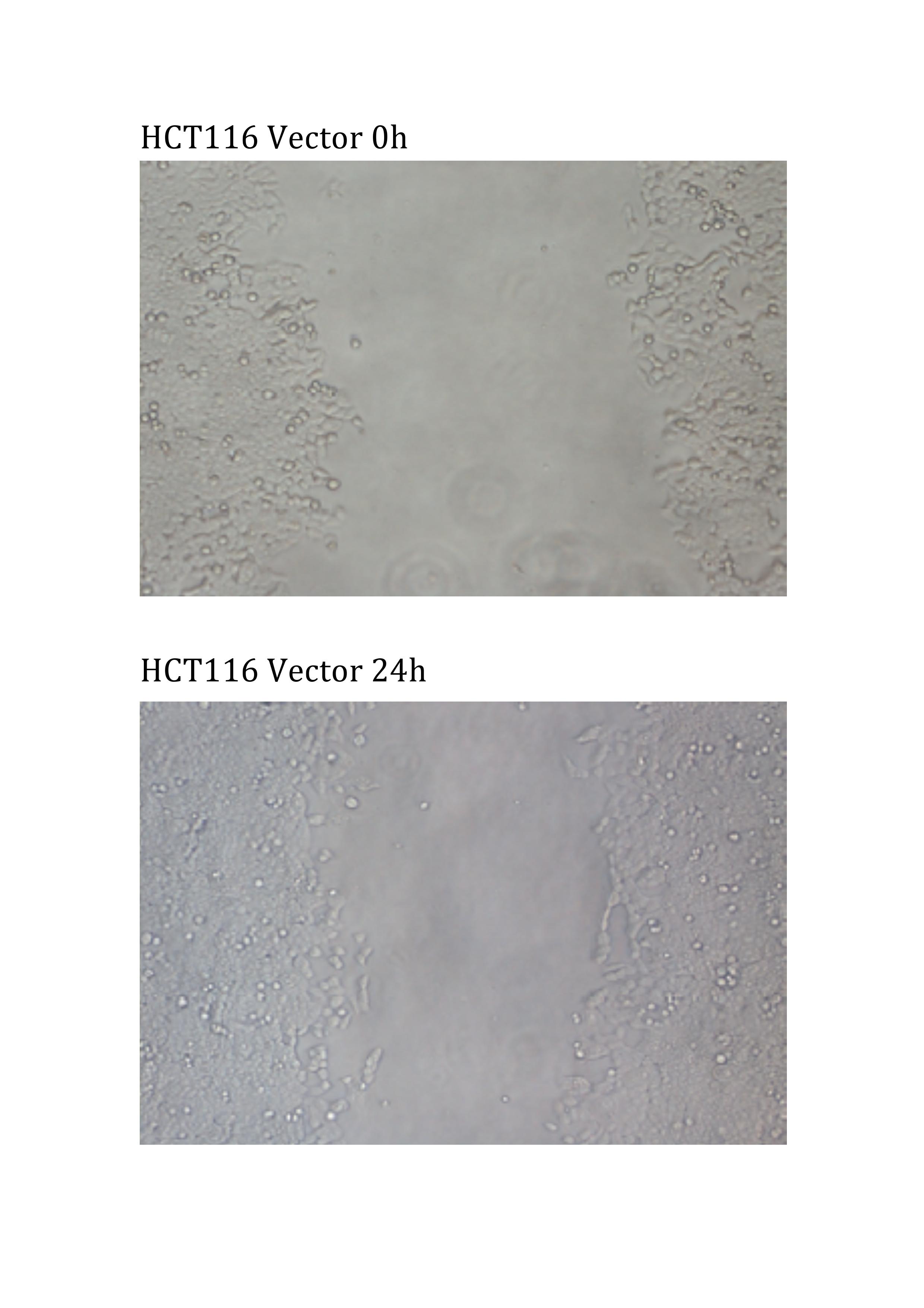

Supplement: Supplementary file 2 — Supplemental files. (ZIP 13000 kb) [file 13046_2018_879_MOESM2_ESM.zip › 0019.jpg]

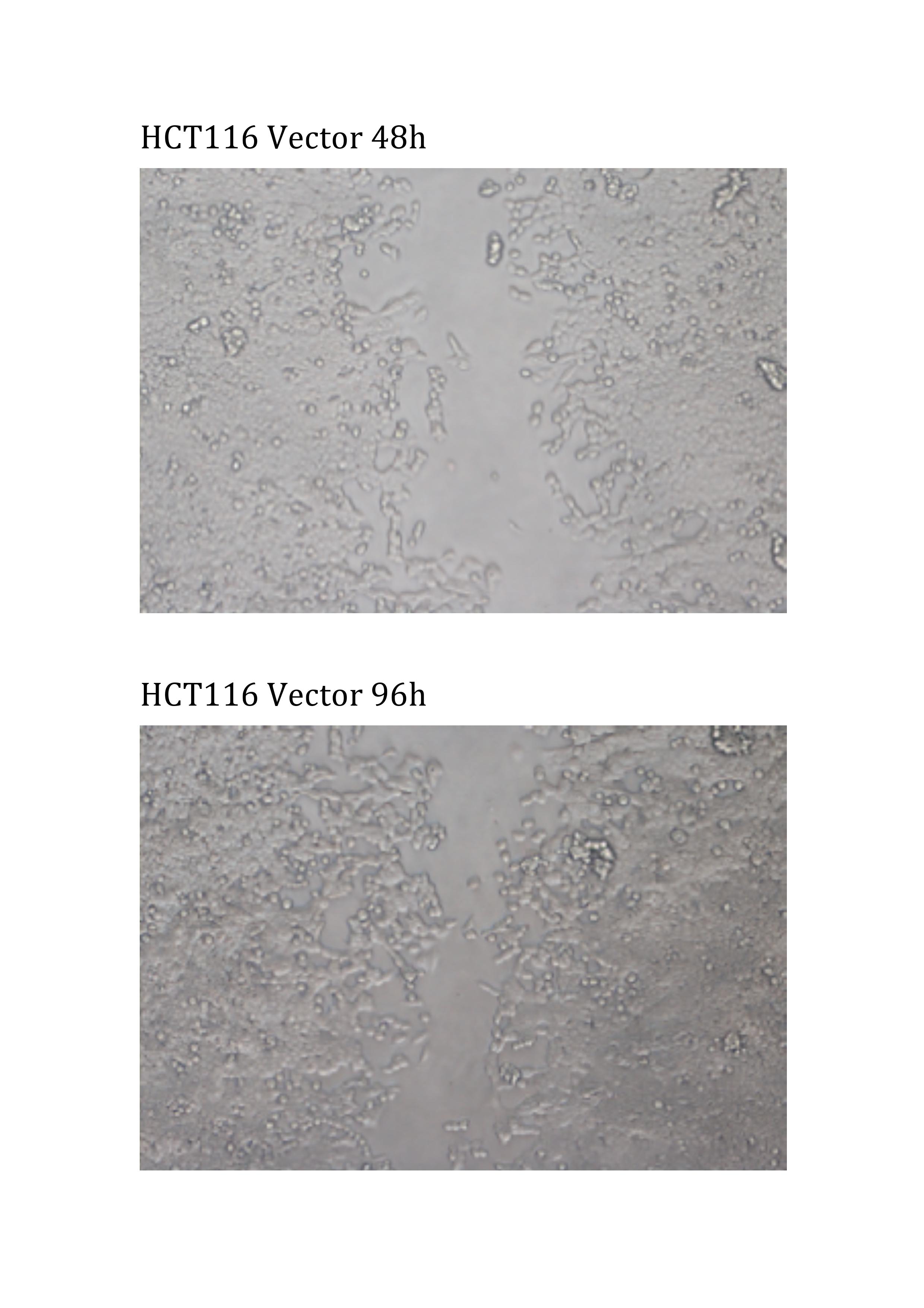

Supplement: Supplementary file 2 — Supplemental files. (ZIP 13000 kb) [file 13046_2018_879_MOESM2_ESM.zip › 0020.jpg]

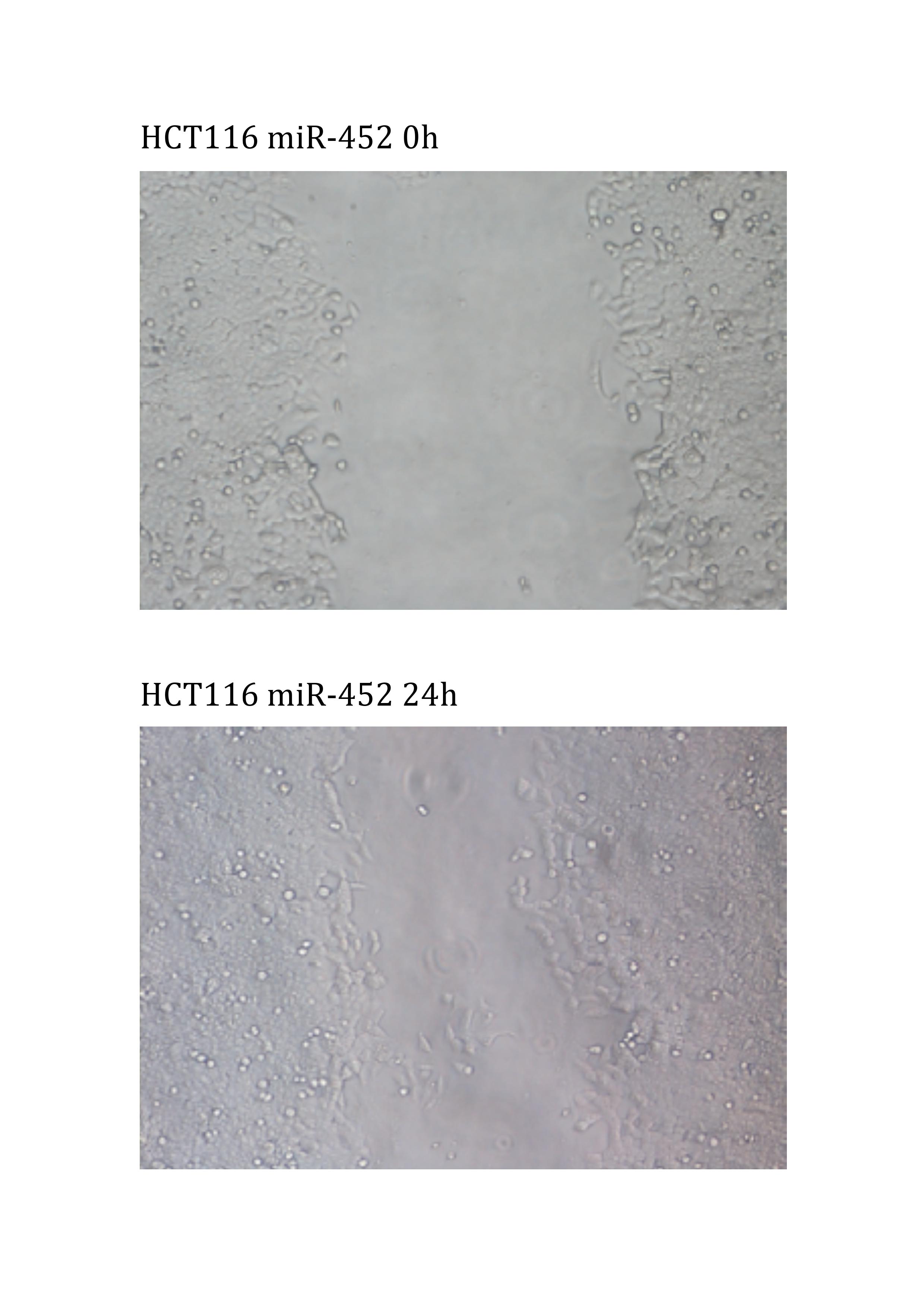

Supplement: Supplementary file 2 — Supplemental files. (ZIP 13000 kb) [file 13046_2018_879_MOESM2_ESM.zip › 0021.jpg]

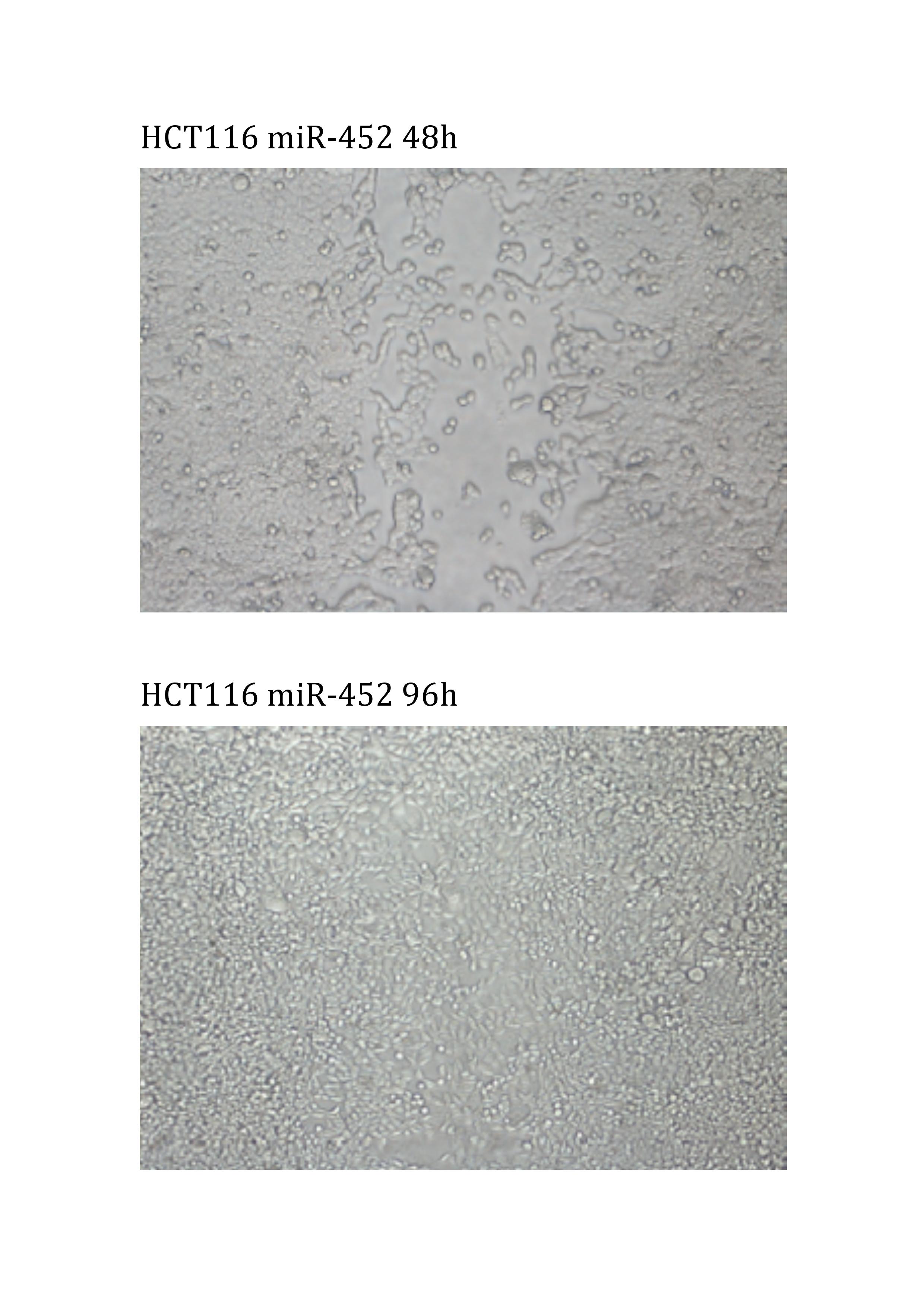

Supplement: Supplementary file 2 — Supplemental files. (ZIP 13000 kb) [file 13046_2018_879_MOESM2_ESM.zip › 0022.jpg]

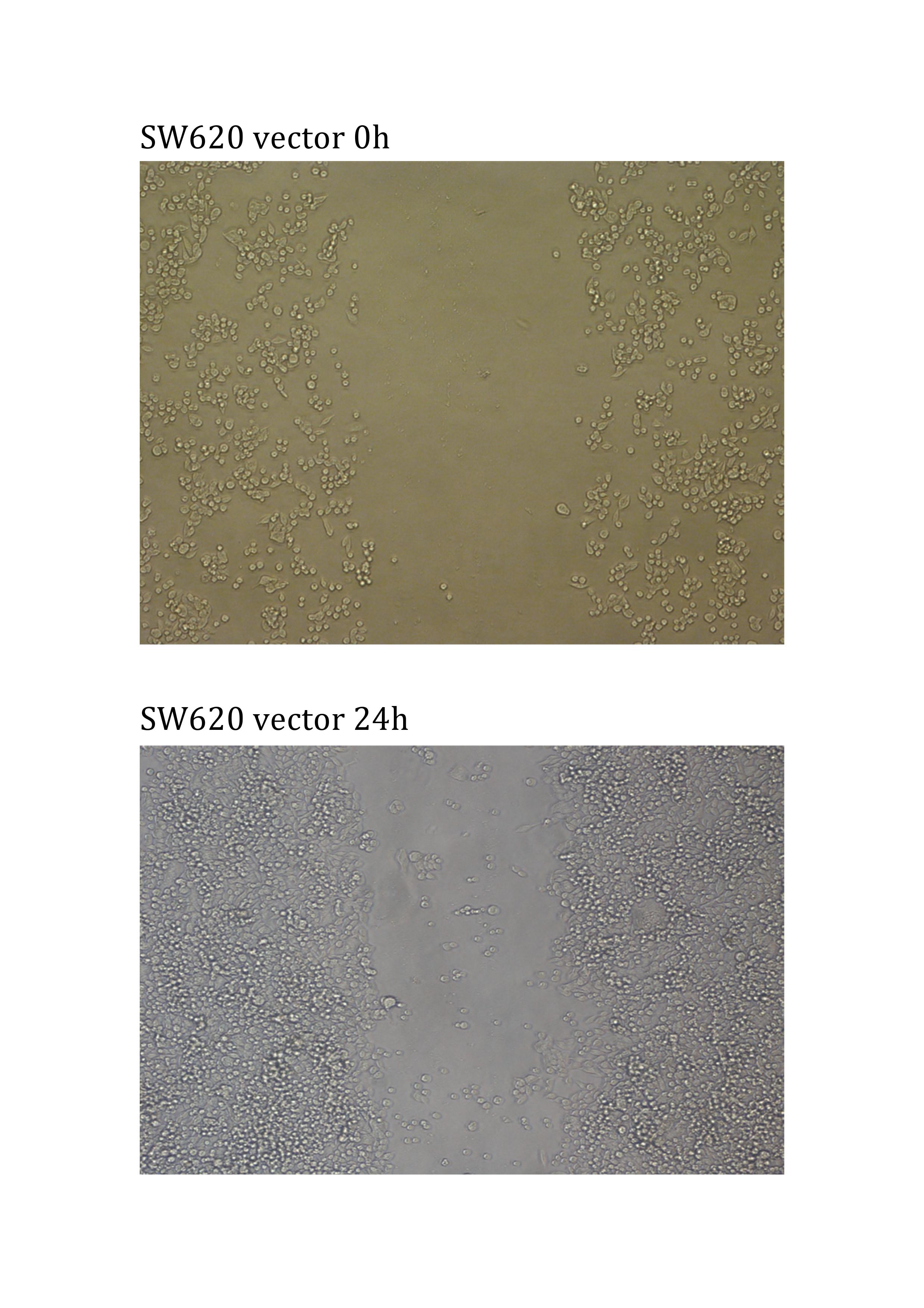

Supplement: Supplementary file 2 — Supplemental files. (ZIP 13000 kb) [file 13046_2018_879_MOESM2_ESM.zip › 0023.jpg]

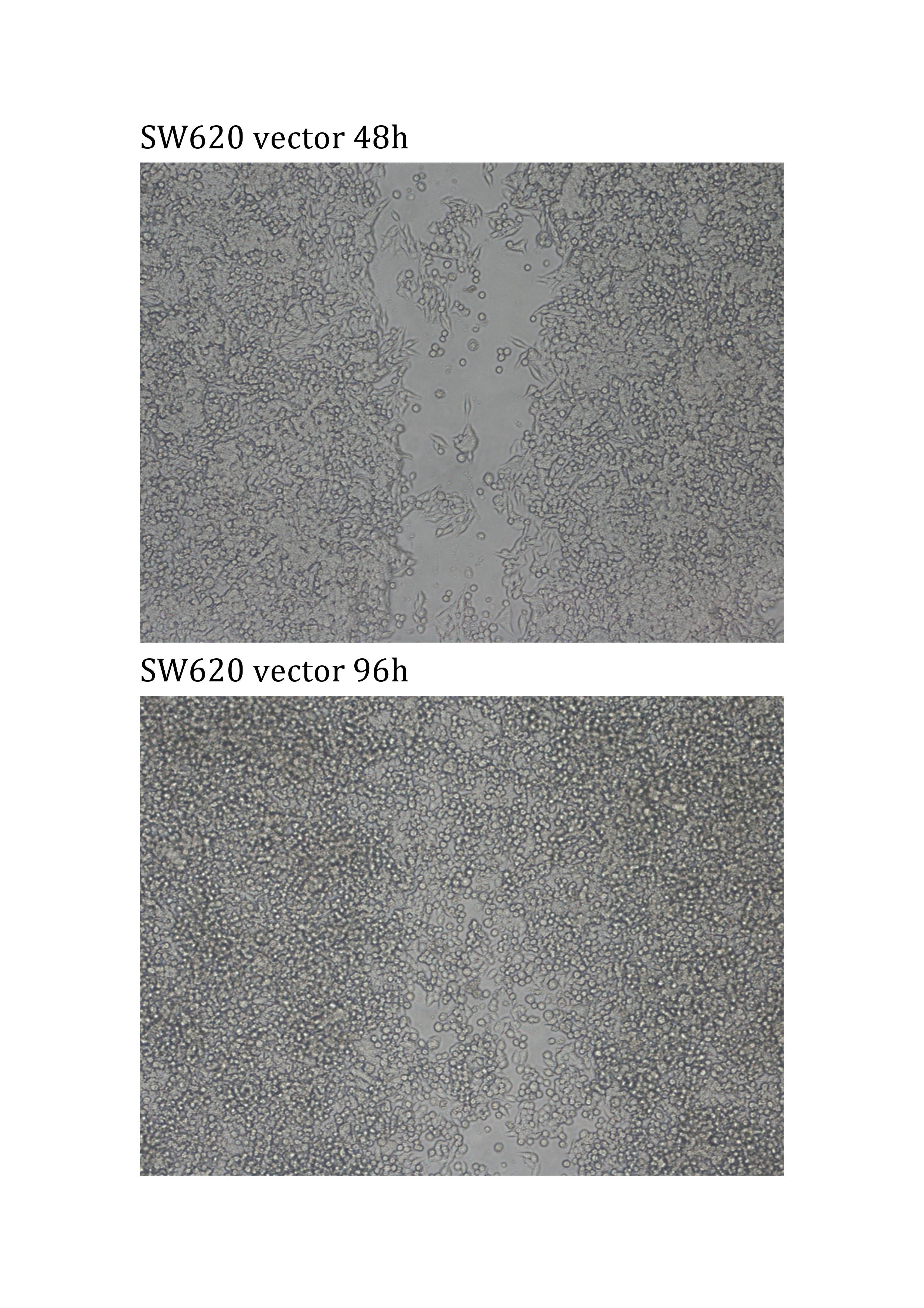

Supplement: Supplementary file 2 — Supplemental files. (ZIP 13000 kb) [file 13046_2018_879_MOESM2_ESM.zip › 0024.jpg]

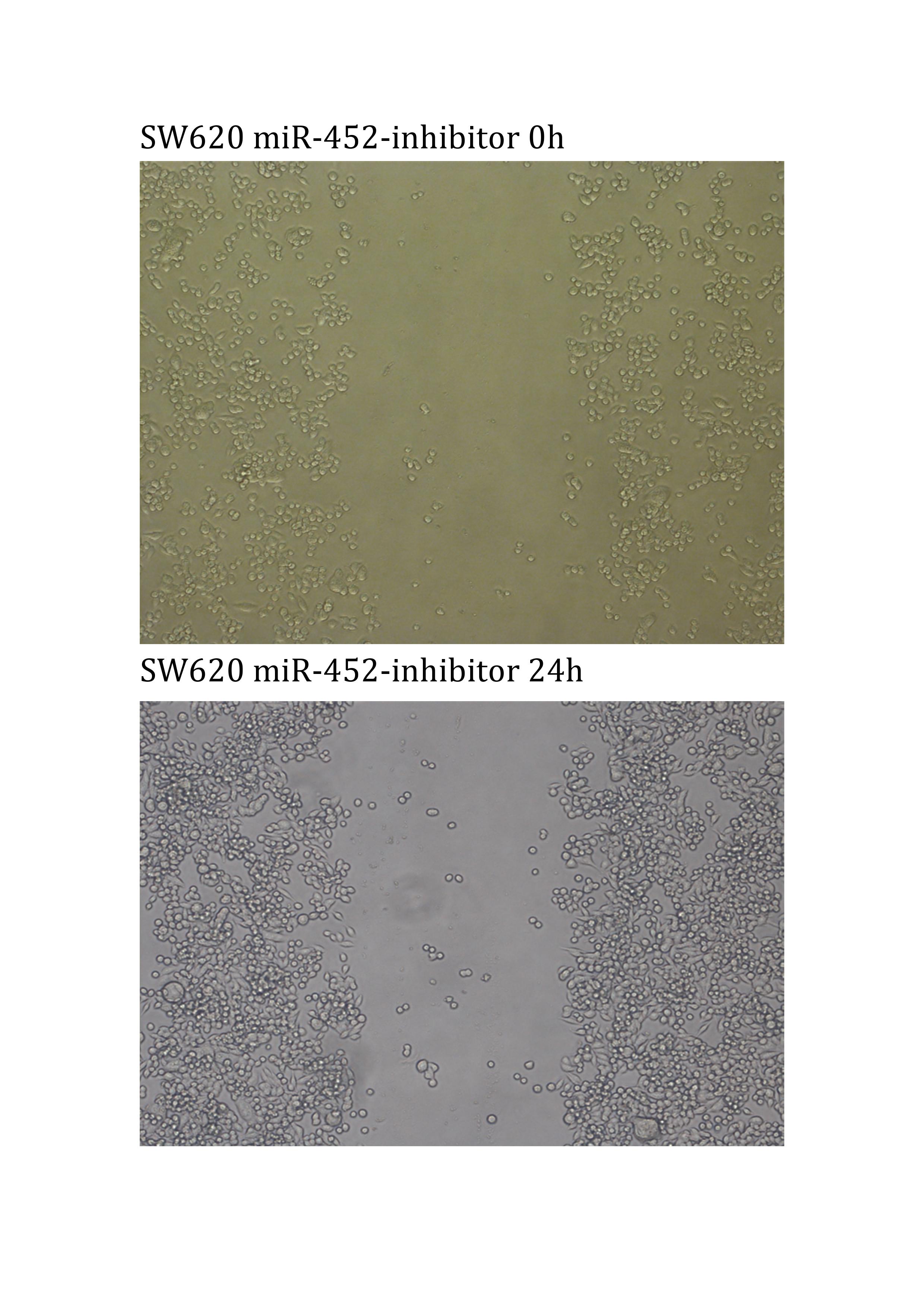

Supplement: Supplementary file 2 — Supplemental files. (ZIP 13000 kb) [file 13046_2018_879_MOESM2_ESM.zip › 0025.jpg]

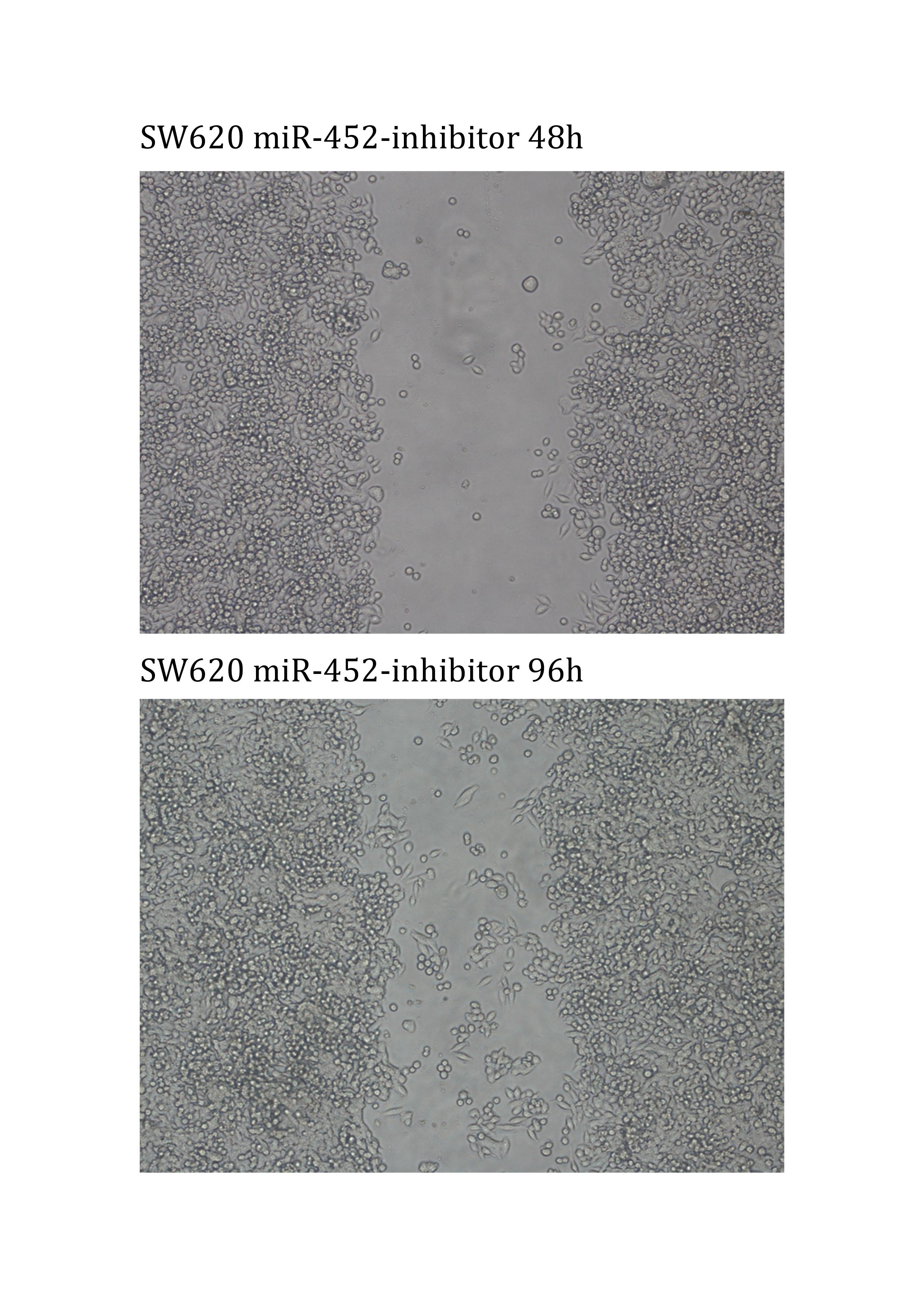

Supplement: Supplementary file 2 — Supplemental files. (ZIP 13000 kb) [file 13046_2018_879_MOESM2_ESM.zip › 0026.jpg]

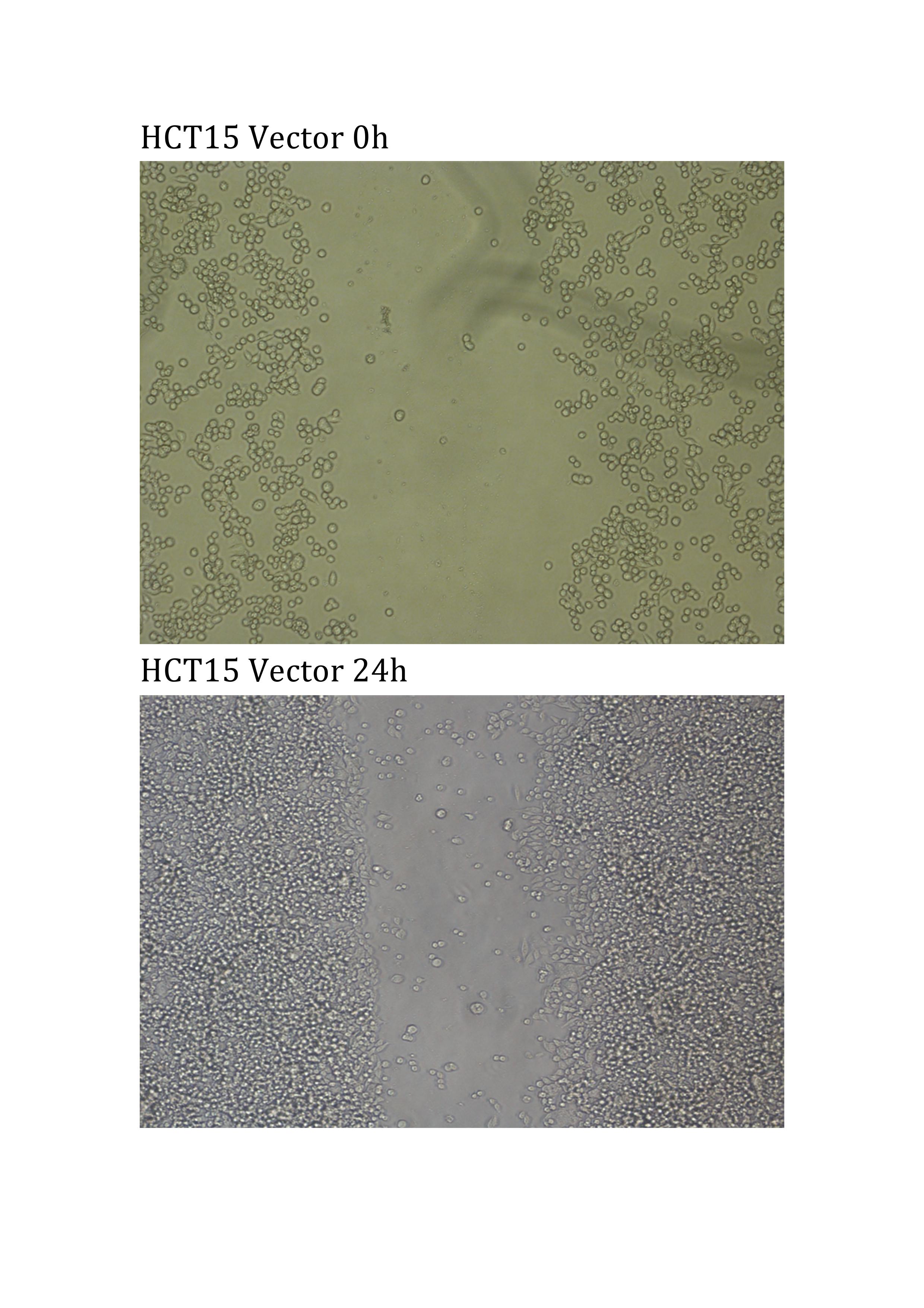

Supplement: Supplementary file 2 — Supplemental files. (ZIP 13000 kb) [file 13046_2018_879_MOESM2_ESM.zip › 0027.jpg]

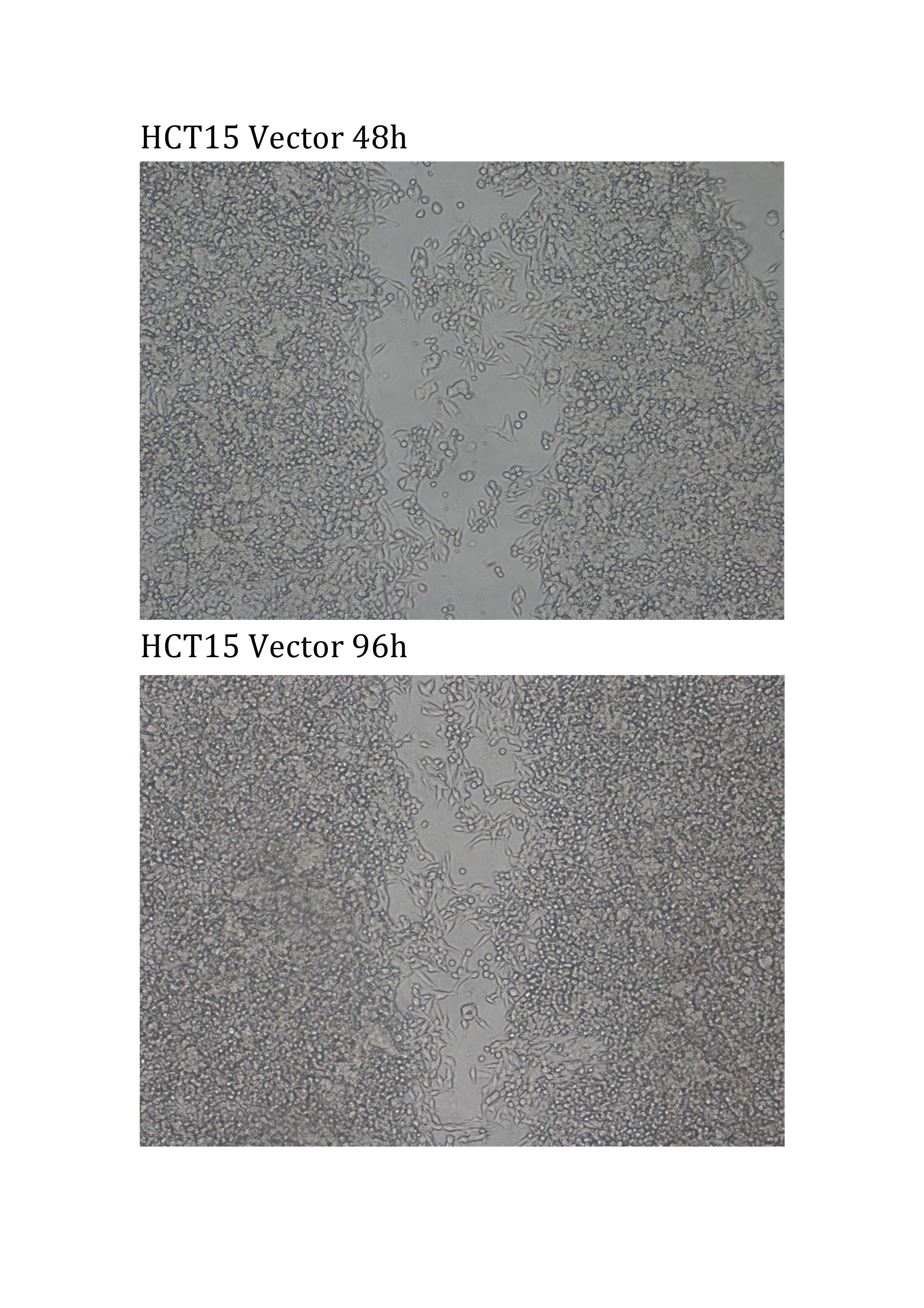

Supplement: Supplementary file 2 — Supplemental files. (ZIP 13000 kb) [file 13046_2018_879_MOESM2_ESM.zip › 0028.jpg]

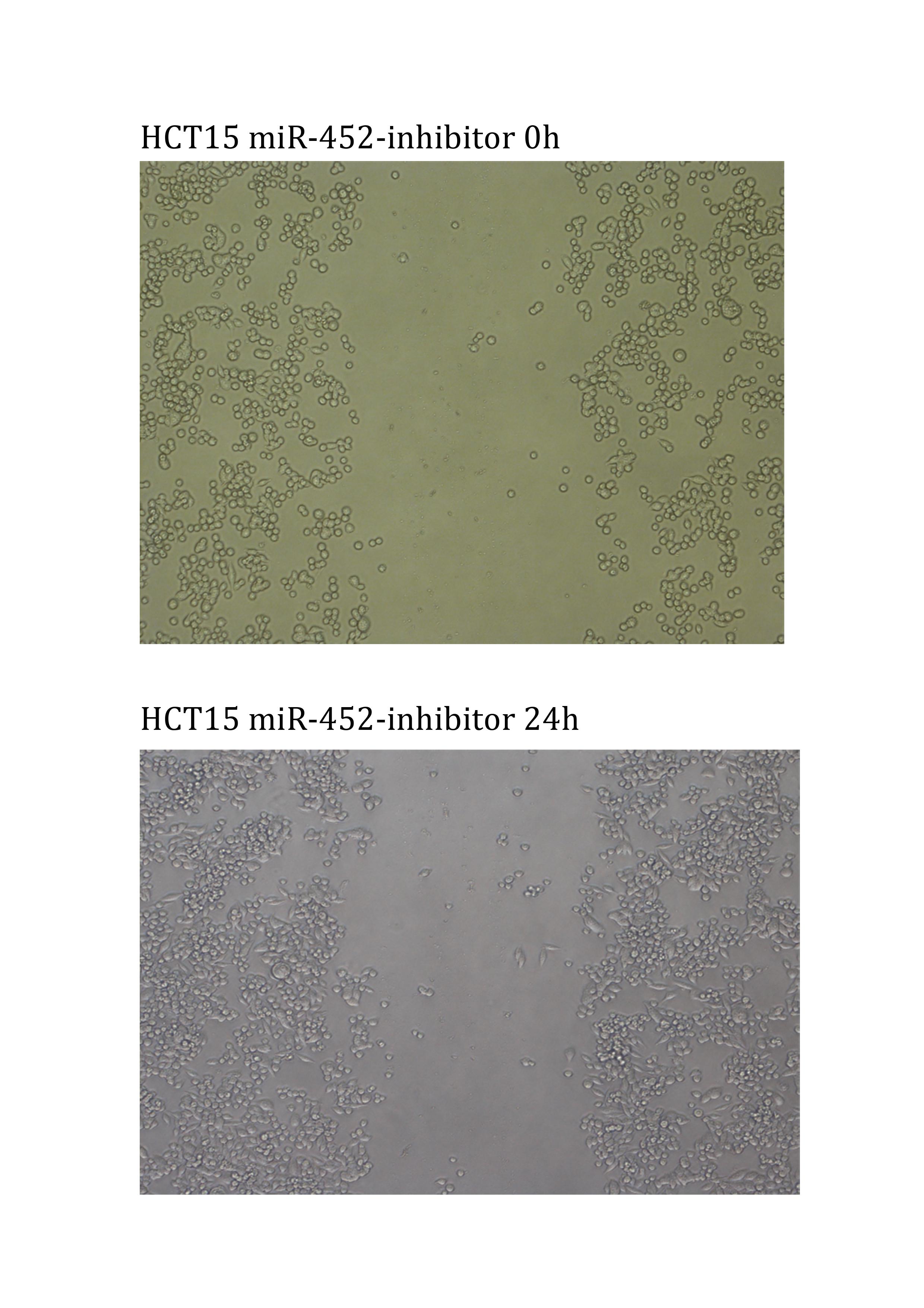

Supplement: Supplementary file 2 — Supplemental files. (ZIP 13000 kb) [file 13046_2018_879_MOESM2_ESM.zip › 0029.jpg]

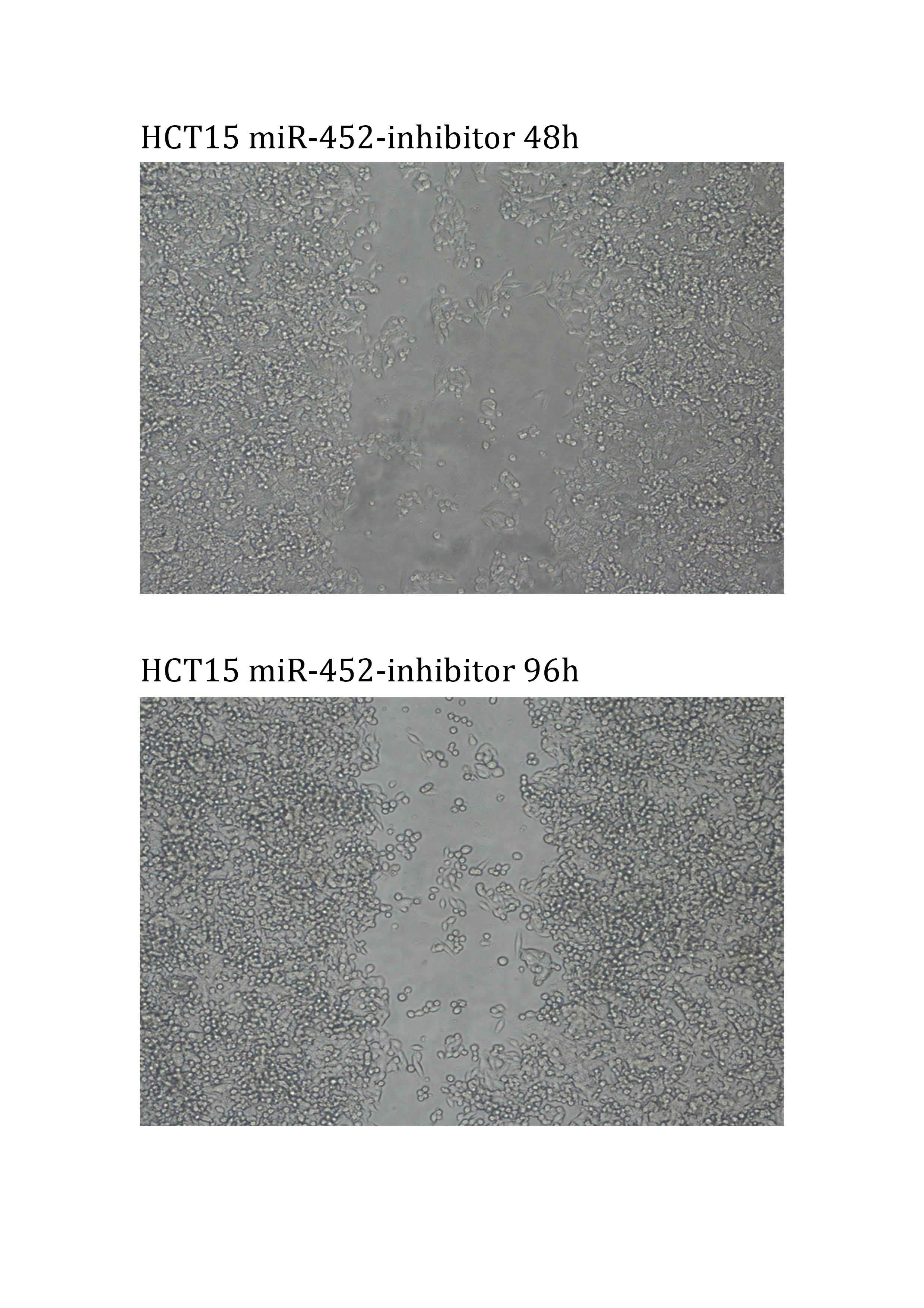

Supplement: Supplementary file 2 — Supplemental files. (ZIP 13000 kb) [file 13046_2018_879_MOESM2_ESM.zip › 0030.jpg]

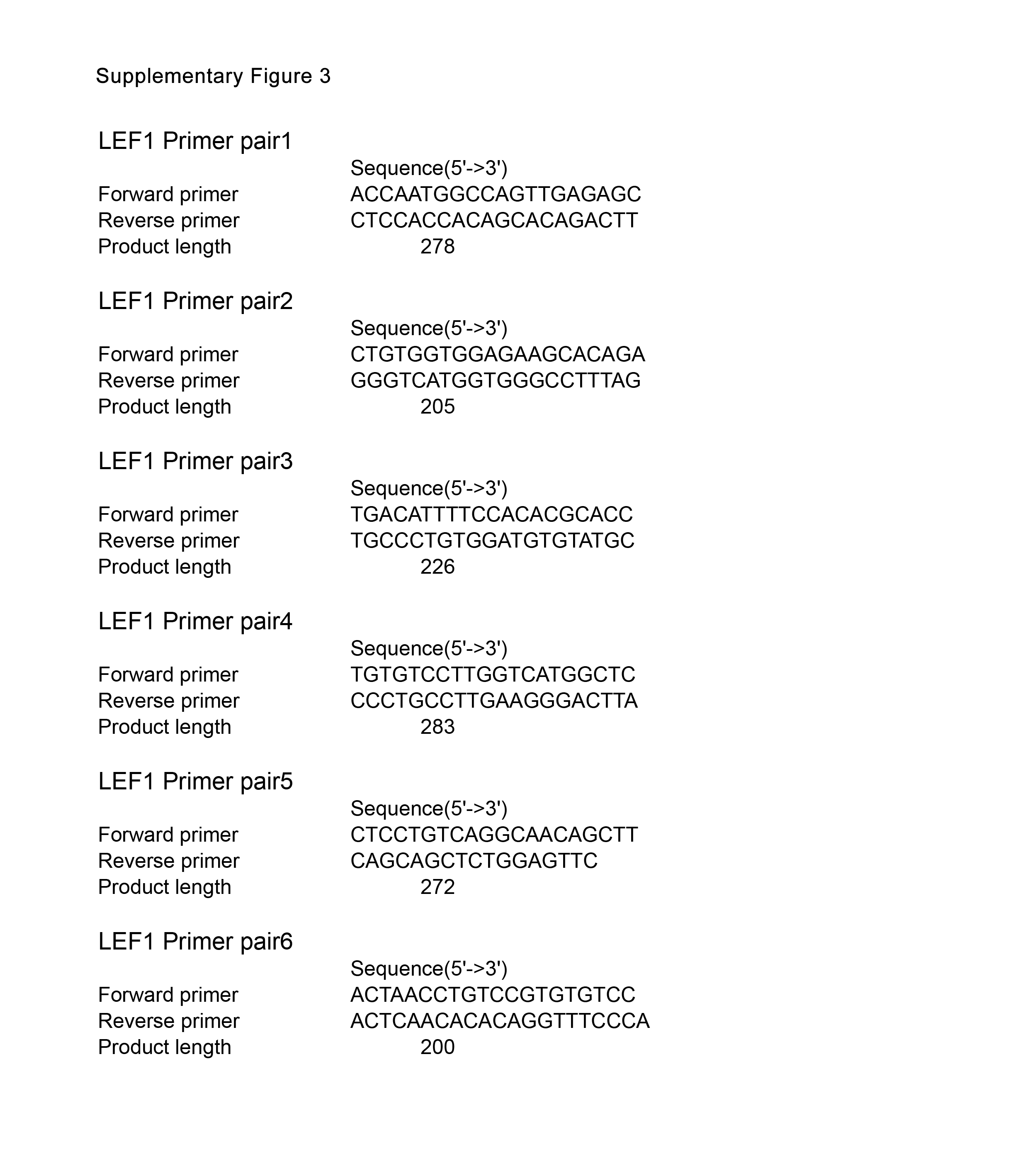

Supplement: Supplementary file 3 — Figure S3. Chip primer sequenced used for quantitative real-time PCR. (TIF 319 kb) [file 13046_2018_879_MOESM3_ESM.tif]

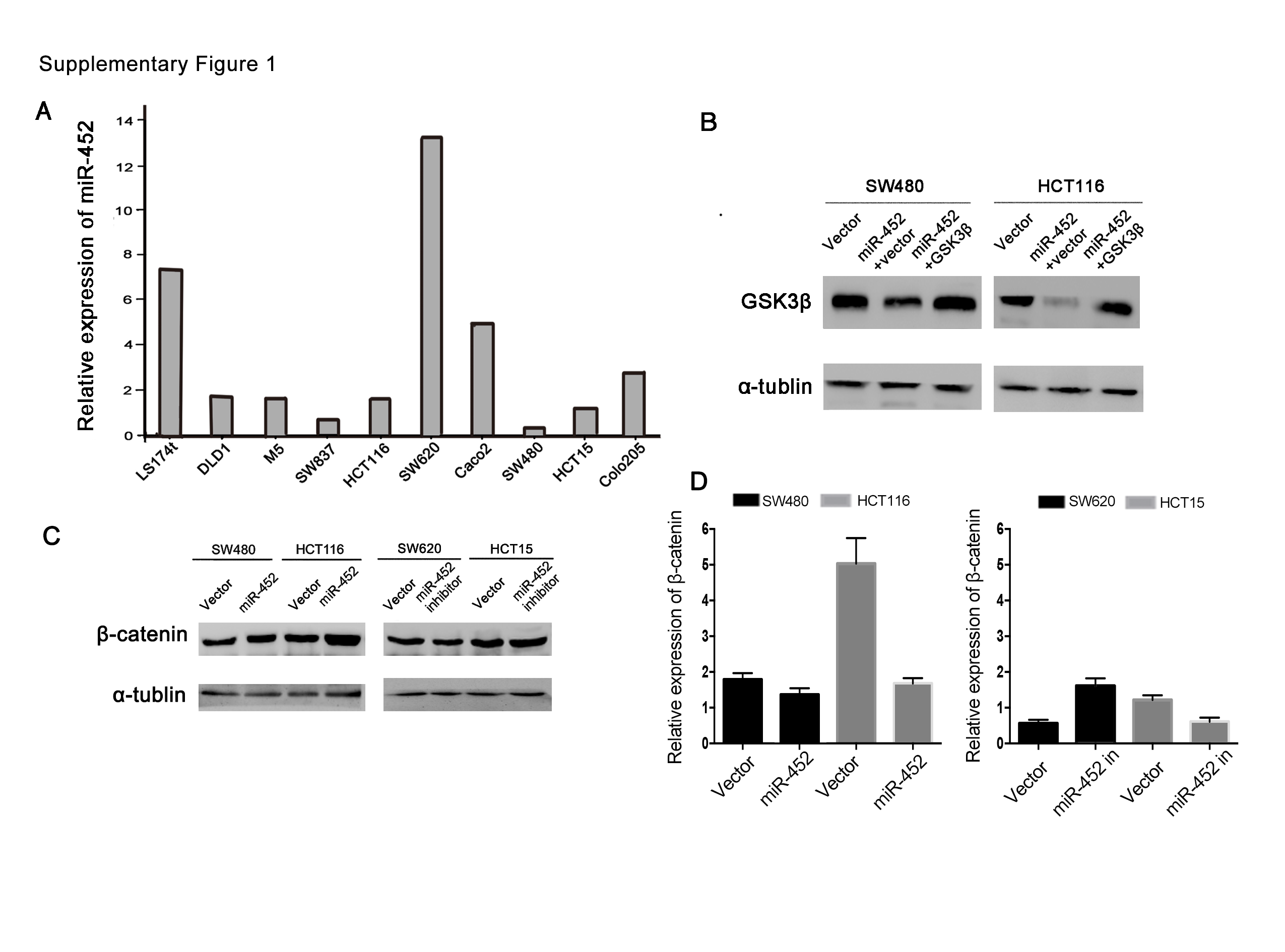

Supplement: Supplementary file 4 — Figure S1. MiR-452 expression in CRC cells and transfection efficency of miR-452/GSK3β. (A) The relative exprssion of miR-452 in 10 colorectal cancer cells by qPCR. (B) Western blot analyses of GSKβ in cell transfected with Vector, miR-452, miR-452/GSK3β in SW480 and HCT116 cells. (C) Western blot analyses of β-catenin in the indicated cells. (D) Real-time quantitative PCR analysis of mRNA relative expression of β-catenin in the indicated cells. (TIF 14145 kb) [file 13046_2018_879_MOESM4_ESM.tif]
